# Interfacial charge distributions in carbon-supported palladium catalysts

**DOI:** 10.1038/s41467-017-00421-x

**Published:** 2017-08-24

**Authors:** Radhika G. Rao, Raoul Blume, Thomas W. Hansen, Erika Fuentes, Kathleen Dreyer, Simona Moldovan, Ovidiu Ersen, David D. Hibbitts, Yves J. Chabal, Robert Schlögl, Jean-Philippe Tessonnier

**Affiliations:** 1 Department of Chemical and Biological Engineering, Iowa State University, Ames, IA 50011 USA; 2 NSF Engineering Research Center for Biorenewable Chemicals, Ames, IA 50011 USA; 3 Fritz Haber Institute of the, Max Planck Society, DE-14195 Berlin, Germany; 4 Center for Electron Nanoscopy, Technical University of Denmark, DK-2800 Kgs Lyngby, Denmark; 5 Department of Materials Science and Engineering, University of Texas at Dallas, Richardson, TX 75080 USA; 6 Department of Chemical Engineering, University of Florida, Gainesville, FL 32611 USA; 7 Institut de Physique et Chimie des Matériaux de Strasbourg, UMR 7504 University of Strasbourg – CNRS, FR-67200 Strasbourg, France

## Abstract

Controlling the charge transfer between a semiconducting catalyst carrier and the supported transition metal active phase represents an elite strategy for fine turning the electronic structure of the catalytic centers, hence their activity and selectivity. These phenomena have been theoretically and experimentally elucidated for oxide supports but remain poorly understood for carbons due to their complex nanoscale structure. Here, we combine advanced spectroscopy and microscopy on model Pd/C samples to decouple the electronic and surface chemistry effects on catalytic performance. Our investigations reveal trends between the charge distribution at the palladium–carbon interface and the metal’s selectivity for hydrogenation of multifunctional chemicals. These electronic effects are strong enough to affect the performance of large (~5 nm) Pd particles. Our results also demonstrate how simple thermal treatments can be used to tune the interfacial charge distribution, hereby providing a strategy to rationally design carbon-supported catalysts.

## Abstract (web summary)

Control over charge transfer in carbon-supported metal nanoparticles is essential for designing new catalysts. Here, the authors show that thermal treatments effectively tune the interfacial charge distribution in carbon-supported palladium catalysts with consequential changes in hydrogenation performance.

## Introduction

The performance of a catalyst, hence the rate and selectivity of the reaction, can be tailored by controlling the electronic structure of its active sites^1, 2^. Common strategies to achieve the desired properties consist in adjusting the composition, crystallographic structure, particle size, and geometry of the active phase^3–5^. In homogeneous catalysis, electron-donating and electron-withdrawing ligands provide an additional route to modulate the electronic structure of metal catalysts^6^. Analogous effects where charge density is transferred between the metal and the support are known in heterogeneous catalysis since 1960s, when Schwab explained these phenomena using solid state physics^7, 8^. However, harnessing these electronic metal-support interactions (EMSI) for the rational design of heterogeneous catalysts has proven to be difficult to implement, specifically due to the reconstruction of studied catalysts under reaction conditions that obscure the desired electronic effects^2, 9, 10^. For example, the performance of Pt-group metal nanoparticles supported on reducible oxides was dominated by the partial reduction of the support followed by migration of suboxide species onto the metal active phase, causing its encapsulation with an impermeable oxide overlayer^10^. Migration of the support onto the metal active phase is widespread for oxide scaffolds and it has created controversies on the possibility to harness charge transfer effects for the rational design of supported catalysts^2, 8–12^. Yet, several examples suggest that strong interactions with the support could enhance the catalytic performance of transition metals as much as the nature of the metal itself^13^.

Carbon supports represent an obvious alternative to oxides to study electronic metal-support interactions and eliminate the challenges associated with atom migration and metal encapsulation^14^. However, a detailed analysis of the literature demonstrates that previous attempts to understand EMSI effects for carbons have failed due to the structural and chemical complexity of the studied materials^15^. Experiments performed on conventional activated carbons and carbon blacks did not permit to decouple electronic, polar, and acid–base interactions, and assess their individual impact on substrate adsorption and activation^16^. The structure-property relationships that link atom hybridization, structure, heteroatom concentration, and functional group composition for the support with electronic structure and catalytic activity of the metal sites are lacking, which are in turn key to establish general rules for rational catalyst design^17, 18^.

Despite the challenges in establishing a fundamental understanding of carbon materials as catalyst supports, they are common in heterogeneous catalysis due to their relative inertness compared with conventional oxides. While it is true that carbons do not participate directly in the reaction being catalyzed, these supports can significantly affect the observed activity by altering the adsorption of the reactants. For example, acid–base interactions between CO_2_ and nitrogen-functionalized carbon supports increased the rate for CO_2_ reduction by almost two fold^19^. In other studies, the functionalization of the carbon support with oxygen moieties altered its hydrophilic–hydrophobic character, which in turn modified the reactant’s adsorption mode and the selectivity of the reaction^20^. Conversely, electronic effects such as charge transfer between the metal and carbon scaffold were not addressed^20–23^, although it is now well-established that heteroatoms located at defect sites alter the electronic properties of graphene and graphenic carbons^24, 25^. Specifically, the electronic perturbations caused by nitrogen and sulphur can be significant as inert carbons became catalytically active as a result of the doping process. Recent reviews dedicated to these carbocatalysts demonstrate that their applications are widespread and span from water electrolysis to desulfurization and organic coupling reactions^15, 26^. The effect of functionalization and doping with oxygen on electronic properties has received significantly less attention, probably due to the abundance of oxygen-containing species on carbons materials stored in air and the relative inertness of these materials. Yet, in the photonics field, oxygen was shown to significantly alter the optoelectronic properties of graphene oxide by modulating its band gap^27^. Therefore, conventional carbon supports bearing oxygen functionalities may exhibit electronic properties that remain to be harnessed for catalytic applications.

Here, we demonstrate that the graphitic character and surface functionalization of model carbon supports alter their interaction with palladium (Pd) nanoparticles and affect their selectivity for the hydrogenation of cinnamaldehyde, a model α,β-unsaturated compound. X-ray photoelectron spectroscopy (XPS) using synchrotron radiation shows a charge redistribution at the Pd–C interface that is consistent with the observed changes in catalytic activity. Combining spectroscopy, microscopy, and chemisorption techniques with density functional theory (DFT) calculations further reveals a direct relationship between the oxygen concentration at the support’s surface, the charge distribution at the Pd–C interface, and the catalytic activity. These results demonstrate that the oxygen functionalities commonly found on carbon-supported catalysts alter both the surface chemistry and electronic properties of the support. The charge depletion layer is large enough to influence the performance of 5 nm Pd nanoparticles although particles of this size are expected to display bulk-like properties. Our results also show that the interfacial charge distribution can be controlled using thermal treatments, thus providing a simple yet powerful method for fine-tuning the performance of the supported metal catalyst.

## Results

### Synthesis of carbon supports with tailored properties

Stacked cup carbon nanotubes (SCCNTs) were strategically chosen as supports due to their reduced structural and chemical complexity compared with conventional activated carbon and carbon black supports. SCCNTs consist of a well-defined tubular structure terminated with a layer of disordered graphenic carbon^28, 29^. This layer offers opportunities to tailor the surface properties through chemical functionalization and high-temperature annealing without altering the support’s morphology, surface area, and porosity. Hence diffusion and mass transfer for the corresponding catalysts are unaffected by these treatments and their performance can be easily compared^28, 29^.

**Fig. 1:**
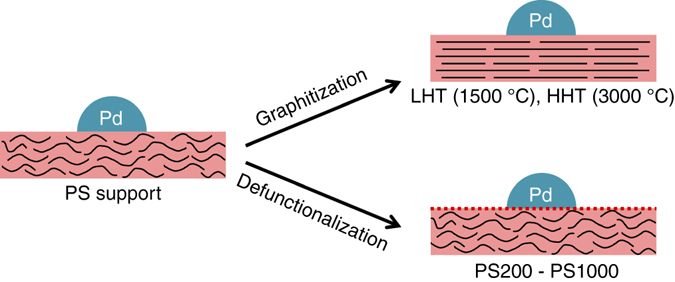
Schematic representation of the studied catalysts. The graphitic character and chemistry of the carbon surface were tailored without altering the textural properties of the support, thus enabling an accurate comparison of the catalytic performance of the studied materials. Two sample series were prepared: Pd nanoparticles were deposited onto supports of increasing graphitic character (PS, LHT, HHT), and PS supports partially defunctionalized through annealing at temperatures ranging between 200 °C (PS200) and 1000 °C (PS1000). The color coding matches the colors used in Fig. 4, where *red* represents the carbon support and *blue* represents the palladium nanoparticles

Two series of samples labeled PS-HHT and PS200-PS1000 were prepared to distinguish the effects of graphitization and surface functionalization, respectively. For the first series, SCCNTs were thermally annealed at 700 °C (PS), 1500 °C (LHT), and 3000 °C (HHT) post synthesis to graphitize the graphenic carbon (Fig. 1)^28^. This process follows the well-established graphitization mechanism demonstrated by Oberlin^30^ (*vide infra*). The samples were subsequently treated with nitric acid to remove any inorganic impurities resulting from the synthesis and/or annealing treatment (Supplementary Fig. 3). Raman spectroscopy and C1s XPS confirmed that the graphitic character of the support increased with the treatment temperature, reaching a quasi-graphitic state at 3000 °C (Supplementary Figs. 1 and 2 and Supplementary Table 1). The treatment with nitric acid simultaneously oxidized the surface of the SCCNTs (Supplementary Table 1). For the second series, the PS sample was partially defunctionalized under inert atmosphere at selected temperatures ranging between 200 and 1000 °C (PS200, PS400, PS600, PS800, PS1000). The PS-PS1000 support series thus obtained presented a decreasing concentration of surface oxygen functionalities as demonstrated by the O1s/C1s ratio obtained from XPS, with minor changes in the graphitic character as confirmed by Raman spectroscopy (Supplementary Table 1). The trends observed for surface functionalization and graphitization with annealing temperature were consistent with previous studies^28, 29, 31^. It is important to note that all the supports displayed uniform textural properties despite the changes in the surface chemistry, which makes them the support material of choice in this work^28^.

### Hydrogenation activity of Pd on SCCNT catalysts

**Fig. 2:**
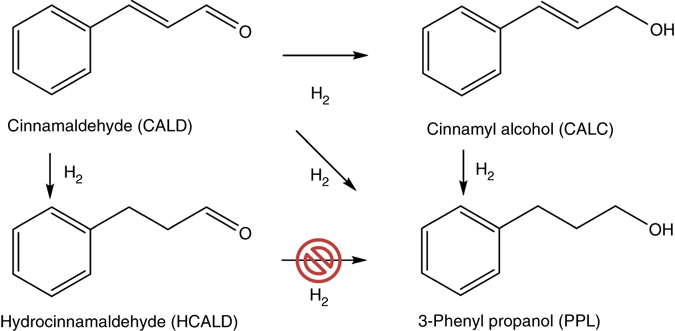
Reaction scheme for the hydrogenation of cinnamaldehyde. Reaction pathways for CALD hydrogenation as observed for Pd/C catalysts at 80 °C, 90 mg ml^−1^ of CALD in dioxane, 100 mg catalyst, and 20 ml min^−1^ hydrogen flow rate at ambient pressure. Only two products were formed under the reaction conditions selected in the present study: HCALD through C=C bond hydrogenation, and PPL through a hydrogenation pathway that does not involve HCALD (i.e., tests performed with HCALD as the reactant showed no conversion, in agreement with Jiang et al. ^35^). CALC and reaction byproducts were not detected, in agreement with the high (> 97%) carbon balance (Supplementary Tables 2 and 3)

The Pd/SCCNT catalysts were prepared by incipient wetness impregnation of the tailored supports using an aqueous solution of palladium nitrate. This precursor was preferred over other salts as its reduction yields Pd nanoparticles free of any organic and inorganic impurities. The use of surfactants to obtain monodisperse particles was not considered either, due to the possible modification of the steric and electronic properties of the metal active sites by the surfactants^32–34^. The obtained catalysts were tested for the ambient pressure and low temperature (80 °C) hydrogenation of cinnamaldehyde (CALD), an α,β-unsaturated aldehyde model compound (Fig. 2, Supplementary Fig. 4). This reaction was selected for its sensitivity to the electronic properties of the metal active phase^35, 36^. Specifically, selectivity towards C=C and/or C=O hydrogenation products was shown to depend on the position of the metal’s d band center^35, 36^, which depends on the nature of the metal and alteration of its electronic properties through, for example, alloying or doping. Therefore, this reaction provides an excellent platform for probing metal-support charge transfers.

CALD conversion can proceed via hydrogenation of the C=C bond (hydrocinnamaldehyde, HCALD), C=O bond (cinnamyl alcohol, CALC), or both (3-phenyl propanol, PPL), as shown in Fig. 2. Pd catalysts exhibit a higher selectivity for C=C bond hydrogenation, which is the thermodynamically favored route for this reaction^37^. However, fluctuations in selectivity were observed depending on the support, even among carbon scaffolds. Although attempts have been made in the past to identify the parameters that control the selectivity to HCALD, the role of key factors such as support polarity, metal particle size, and reaction conditions remain intertwined^21, 23^.

**Fig. 3:**
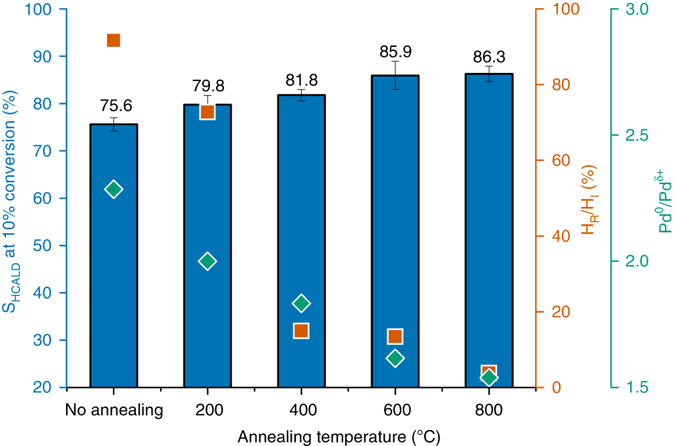
Effect of carbon supports on catalytic performance. The *bar graph* shows the selectivity to HCALD at 10% CALD conversion for Pd supported on the PS support and on PS defunctionalized at 200–800 °C (*blue*). The values correspond to the average selectivity calculated for three independent experiments ± the corresponding standard deviation (s.d.). The selectivity to HCALD was found to increase with the annealing temperature although the average particle size, metal loading, and normalized reaction rate were within error for the five samples. This trend was consistent with changes in the active metal phase revealed by hydrogen chemisorption (ratio of reversibly to irreversibly chemisorbed hydrogen H_R_/H_I_, *orange*) and by XPS (ratio of the Pd 3d5/2 peaks for the metal to δ + phase, Pd^0^/Pd^δ+^, *green*)

Significant differences in selectivity were observed for the PS-PS800 sample series (Fig. 3, Supplementary Fig. 5). The observed increase in selectivity to HCALD with the defunctionalization temperature is consistent with previous studies that suggested a correlation exists between selectivity and surface polarity^21, 36^. Non-polar surfaces were proposed to favor the planar (i.e., flat) adsorption mode due to interactions between CALD’s benzene ring and the delocalized pi system of the carbon support, yielding HCALD through preferential hydrogenation of the C=C bond. Conversely, polar surfaces were proposed to favor adsorption through the terminal carbonyl functionality, thus enhancing the selectivity to PPL (on Pd) and CALC (on Pt)^21, 36^. While this interpretation is supported by in situ FTIR^36^, it remains unclear whether the adsorption mode governs the selectivity of the reaction or, instead, is the consequence of a preferential CALD adsorption through the alkene or carbonyl functionality depending on the electronic configuration of the metal. In addition, many studies did not consider the possible contribution of artifacts—variations in the metal loading, particle size, and mass transfer limitations—to the observed trends.

### Effect of Pd distribution and mass transfer

Despite the differences in surface oxygen concentration (Supplementary Table 1) and, thereby, in interactions between the carbon surface and Pd precursor, elemental analysis showed a consistent Pd loading of 3.7 ± 0.5 wt% for the PS-PS800 series (Supplementary Table 4). Further, similar particle sizes of 5.0 ± 0.2 nm were measured by hydrogen chemisorption using the double isotherm method (Supplementary Table 5). X-ray diffraction (XRD) and transmission electron microscopy (TEM) provided slightly larger particle sizes, in the order of 5.6 ± 1.3 nm (PS sample) and 6.9 ± 2.7 nm, respectively (Supplementary Table 5). The values obtained were within the error of measurements of the different techniques as observed in other studies as well^38^. The multiple techniques used here allowed us to minimize the limitations and errors intrinsic to each technique and thereby their impact on the interpretation of our results. The similar loading, particle sizes, and particle shapes obtained for all the catalysts indicate that the trends in catalytic performance shown in Fig. 3 are not a result of particle size or shape effects (Supplementary Figs. 5, 7 and 8)^35^. In addition, the trends are not a result of differences in mass transport either as the size, aspect ratio, surface area, and porosity of the supports were not altered by the functionalization and annealing treatments. The Pd/SCCNTs catalysts consist of mesoporous nanotubes^28, 29^, with all the metal particles located on the outer surface and inside the 30 nm mesopore that extends along the main axis of the tube (Supplementary Figs. 6 and 7). In addition, all the selectivities were reported at iso-conversion, which is critical for sequential reactions and also to ensure that the periodic withdrawal of samples has a minimal impact on the selectivity of the catalysts^39^, in coherence with the chemical reaction engineering principles^20, 21^.

### Support effects on the electronic structure of palladium

**Fig. 4:**
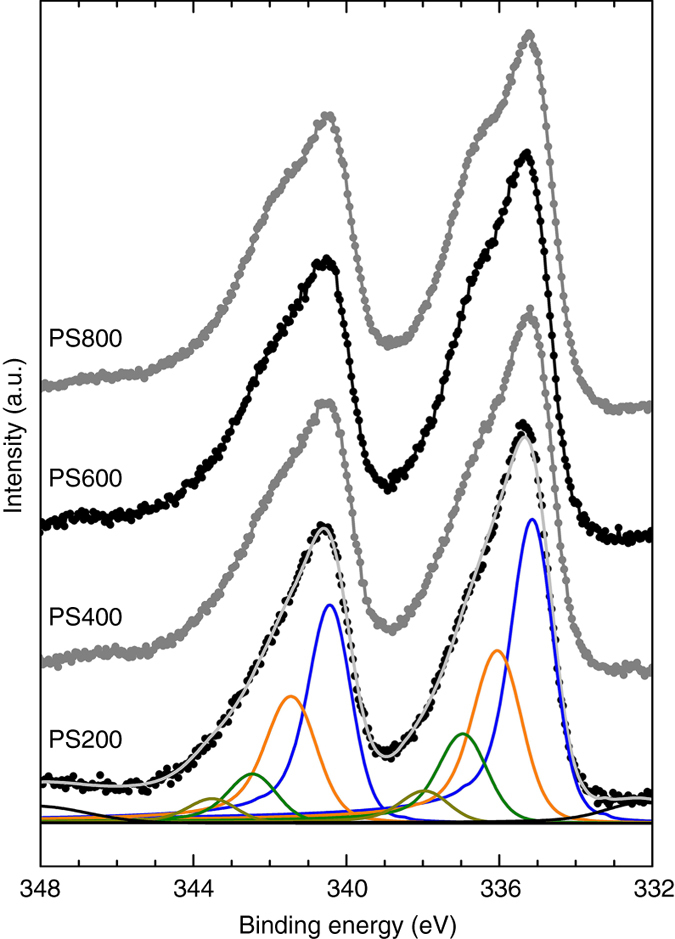
Lab-source XPS spectra for Pd/PS200 to Pd/PS800 catalysts. The deconvolved peaks were assigned to Pd metal at 335.1 eV (*blue*), Pd^δ+^ at 335.95 eV (*orange*), and Pd oxides at 336.9 eV and 337.85 eV (*green*). The increasing contribution of Pd^δ+^ with treatment temperature largely contributes to the shoulder that appears in the overall spectra between 336 and 337 eV

XPS using both lab and synchrotron radiation was used to examine the effect of the carbon support on the electronic structure of the metal active phase (Fig. 4 and Supplementary Figs. 9 and 10). These two X-ray sources provide photons with very different energies, thus allowing for a depth profiling of the catalysts. Spectra using synchrotron radiation were acquired with photon energies of 1020 eV (survey), 680 eV (O1s), 480 eV (Pd3d) and 425 eV (C1s), and with a spectral resolution of ~ 0.3 eV. The kinetic energy of the generated photoelectrons corresponds to a mean free path of ~ 6 Å and a total information depth of ~ 2 nm, that is, 95% of all detected electrons originated from 3λ^40^. In contrast, the laboratory XPS instrument used a Mg source to generate photons of 1253.688 eV (Mg Kα1) and 1253.437 eV (Mg Kα2), and offered an energy resolution of ~ 0.5 eV. The ability to acquire spectra with higher energy resolution and better surface sensitivity using synchrotron radiation ensured that the measured signals are representative of the support’s surface and catalytically active palladium atoms^28, 41^.

**Fig. 5:**
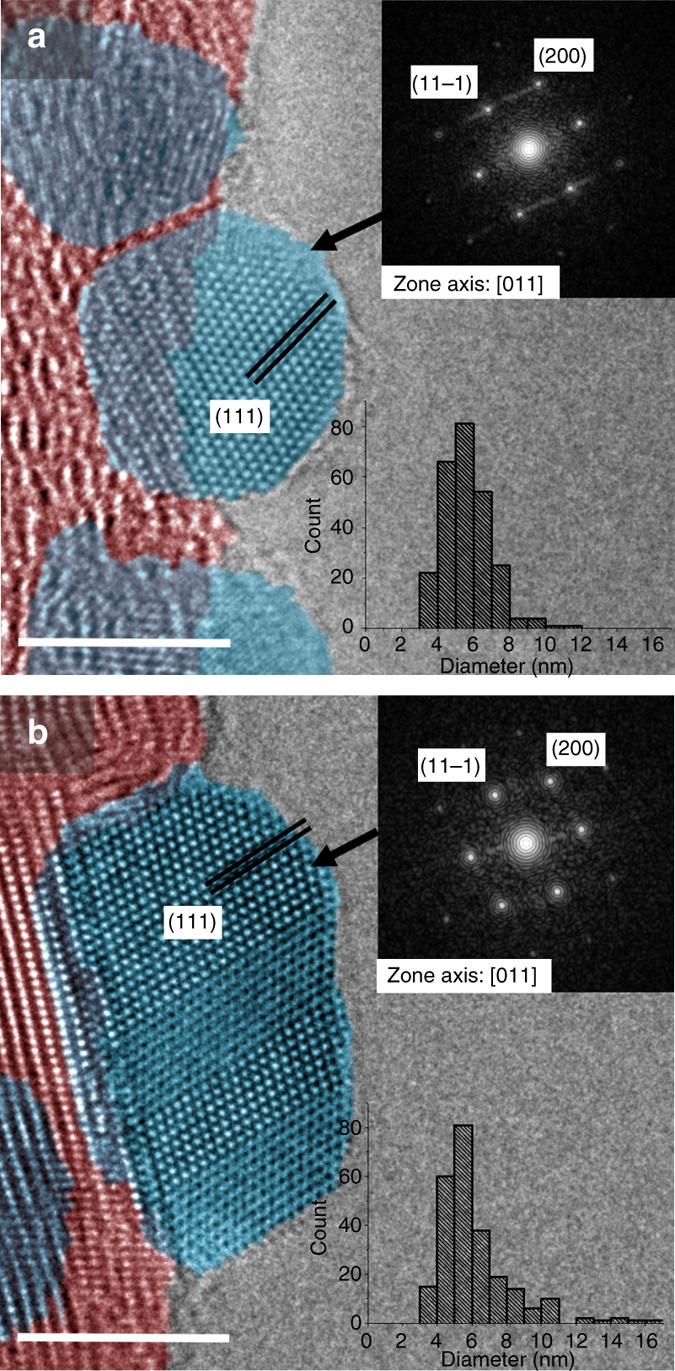
Aberration-corrected TEM. The micrographs acquired for the **a** Pd/PS and **b** Pd/HHT catalysts provide an atomic-resolution view of the Pd nanoparticles (*blue*) and of their interface with the carbon support (*red*). The *insets* show the fast Fourier transform results and the particle size distribution. The *scale bars* in the micrographs **a**, **b** represent 5 nm

The C1s and O1s spectra were analyzed using the method developed by Blume et al.^41^ for carbon nanomaterials. This method notably allows to distinguish contributions from sp^2^ carbon atoms in a graphene-like honeycomb environment, atoms at point-like defects, and atoms in highly disordered environments such as edges and large vacancies (Supplementary Fig. 2). The evolution of the three signals follows a pattern consistent with the graphitization mechanism proposed by Oberlin^30^. As temperature is increased to 800 °C, oxygen-containing functional groups are thermally decomposed to CO, CO_2_, and H_2_O following a sequential process that has been documented extensively in the carbon field. This process is accompanied by a re-aromatization of the corresponding sites, hence the steady decrease in point-like defects shown in Supplementary Fig. 2. The concentration of disordered carbon remains constant during defunctionalization as temperatures in excess of 1000 °C are required to initiate the long-range reorganization and graphitization of graphenic carbons. Graphitization becomes evident from XPS and Raman spectroscopy above 1000 °C and a quasi-graphitic state with stacked graphene sheets extending over tens of nanometers is reached after annealing at 3000 °C (Fig. 5, Supplementary Table 1, Supplementary Figs. 2 and 8). The agreement between Raman spectroscopy and synchrotron XPS, which are two techniques with very different information depths, supports that the observed trends hold for the carbon surfaces in direct contact with the Pd nanoparticles.

In agreement with the literature, Pd was observed in its various oxidation states, including contributions corresponding to bulk Pd metal (335.1 eV) and surface oxides (336.9 and 337.85 eV). The presence of an additional prominent signal at 335.95 eV was required to fit the spectra and largely contributed to the shoulder visible in Fig. 4. This binding energy is inconsistent with the values reported in the literature for Pd^1+^ (~ 335.5 eV) and Pd^2+^ (336.3–336.9 eV, assigned to PdO)^42^. Therefore, we attributed this contribution to electron-depleted palladium atoms (Pd^δ+^) resulting from a charge transfer between the metal and the carbon support. Other groups have also reported on the presence of an additional Pd^δ+^ contribution in the spectra of commercial Pd/C catalysts but they did not comment on the origin of this contribution^38, 43^. Interestingly, in our work, the intensity ratio Pd^0^/Pd^δ+^ was found to decrease with increasing annealing temperature for the Pd/PS-PS800 catalysts series (Fig. 3 and Supplementary Table 6), suggesting a direct correlation between support properties and electronic structure of the Pd phase. These metal-support interactions are significant as they alter the metal’s core (3d) electrons. This effect is comparable to a change in oxidation state. Therefore, a measurable impact on the valence electrons, hence on the catalytic performance, is expected. This hypothesis was confirmed by the consistency in trends for Pd^δ+^, HCALD selectivity, and annealing temperature for catalysts supported on PS-PS800 scaffolds (Fig. 3).

The treatment at 1000 °C marks the transition from defective graphenic carbons to highly ordered supports, as discussed erlier in this section. Starting at 1000 °C, the surface oxygen concentration further drops from 9 to 3% (Supplementary Table 1) and the structural defects are annealed until nearly all carbon atoms are in a graphitic environment (Supplementary Fig. 2). Interestingly, the trends observed for the Pd/PS-PS800 series diverged for supports annealed at temperatures above 1000 °C. HCALD selectivity reached a plateau for Pd/PS1000 and slightly decreased for the Pd/HHT sample, again following the same trend as the Pd^δ+^ contribution (Supplementary Tables 2 and 6). Therefore, it appears that an optimal support structure exists and tailoring the defect density and surface functionalization offers a powerful route to fine-tune the performance of Pd/C catalysts. It was previously proposed that defects in graphenic carbons provide anchoring points to disperse and stabilize Pd nanoparticles. While certainly true, our results suggest that additional effects must be considered to synthesize catalysts with optimal performance. Interestingly, deviation from the trends observed for the Pd/PS-PS800 series occurs when the support reaches a graphitic state and gains semi-metallic properties^44^. This observation is consistent with a charge transfer from Pd nanoparticles to the carbon support for which the magnitude of charge transfer depends on the defect structure, and thereby electronic properties of the carbon surface.

### Support effects on adsorption properties of palladium

Although there are converging indications for charge transfers at the Pd–C interface, the detected Pd^δ+^ phase may also be due to non-stoichiometric carbides or oxides. The formation of sub-surface carbon is thermodynamically favorable for 5 nm Ni and Pd nanoparticles and can occur at the metal-support interface through abstraction and diffusion of carbon atoms from the defective support^31, 45^. Therefore, we investigated the possible formation of oxides and carbides by XRD, aberration-corrected TEM, and XPS. The results revealed that this hypothesis does not hold in the present study as no carbon was inserted in the Pd lattice of the studied samples. Specifically, the lattice spacings determined by XRD and TEM image analysis were fully consistent with bulk Pd metal (Pd^0^). No dissolution of carbon in the metal nanoparticles was observed at the interface either (Fig. 5). Larger *d* spacings were measured for the top 2–3 Pd layers but were attributed to the formation of Pd oxides upon sample storage in air, in accordance with the Pd^2+^ contribution in the XPS spectra.

While the electron micrographs did not reveal any distortion of the metal lattice at the Pd–C interface, the images suggest more intimate interactions between the Pd particles and the defective PS carbon support (Fig. 5a) than with the graphitic HHT scaffold (Fig. 5b). Pd atoms at the interface follow the curvature of the wrinkled carbon layers, suggesting that they bind to topological defects and to heteroatoms decorating the amorphous sp^3^ carbon and/or vacancies. In contrast, a defect-free grain boundary between the graphitic surface of the HHT support and Pd metal is visible in Fig. 5b.

Hydrogen isotherms were measured to probe any differences in H_2_ binding and/or spillover that may arise from changes in the electronic structure of Pd surface atoms. Each sample was first reduced in situ and evacuated at 350 °C to prevent any artefacts due to surface oxides^46^. Two isotherms were successively recorded (double isotherm method) to probe the reversibility of the chemisorption process (Supplementary Fig. 11). The ratios of the reversibly (H_R_) to irreversibly bound hydrogen (H_I_)–H_R_/H_I_–(Supplementary Fig. 12 and Supplementary Table 7) were coherent with the catalytic results (Fig. 3, Supplementary Tables 2 and 3), graphitic character, and support functionalization, which was determined by Raman spectroscopy (Supplementary Table 1) and XPS (Supplementary Fig. 2). The higher reversibility for supports with higher surface oxygen concentration has been documented and it was assigned to ligand-like electron donating/withdrawing effect of the support’s oxygen-containing functional groups^47, 48^. While this effect was only discussed for small (~ 2 nm) nanoparticles, our results demonstrate that similar trends hold for larger nanoparticles (~ 5 nm) that would otherwise be expected to demonstrate bulk-like properties^47, 49^. Our combined observations from hydrogen isotherms and XPS provide experimental evidence that supports electron transfer from the metal to the carbon surface.

Hydrogen spillover was further investigated using high-pressure infrared spectroscopy (HP-FTIR)^47, 50, 51^. All samples, both with and without Pd, showed signals characteristic of CH_2_ symmetric and asymmetric stretching modes when measured in vacuum (Supplementary Fig. 13). These bands were assigned to the saturation of the graphenic edges with hydrogen during the reduction step at 400 °C. Introducing hydrogen gas in the cell had no effect on the spectra, which suggested that hydrogen spillover is negligible at low pressure. Spillover was observed for the Pd/SCCNT samples only above a threshold pressure of 7 bar, similar to previous investigations performed on Pd/C (Supplementary Fig. 14)^47, 50, 51^.

As the differences in reversibility for hydrogen chemisorption cannot be explained by spillover when the pressure is low, we examined the possible contribution of hydrogen absorption and the formation of a Pd β-hydride phase^52^. Palladium hydrides have been studied extensively and the conditions for their formation have been established. Thermodynamically, the formation of palladium β-hydride depends on the hydrogen partial pressure, temperature, and Pd particle size^53^. Herein, the low-hydrogen solubility in dioxane, the selected reaction conditions (80 °C, ambient pressure), and the interactions with the carbon support make any contribution from the β-hydride phase to the observed reactivity trends for Pd/SCCNT unlikely^37, 54–56^. This conclusion is further supported by our XPS experiments as the Pd^δ+^ contribution was observed in vacuum.

### Theoretical evidence for charge transfer

**Fig. 6:**
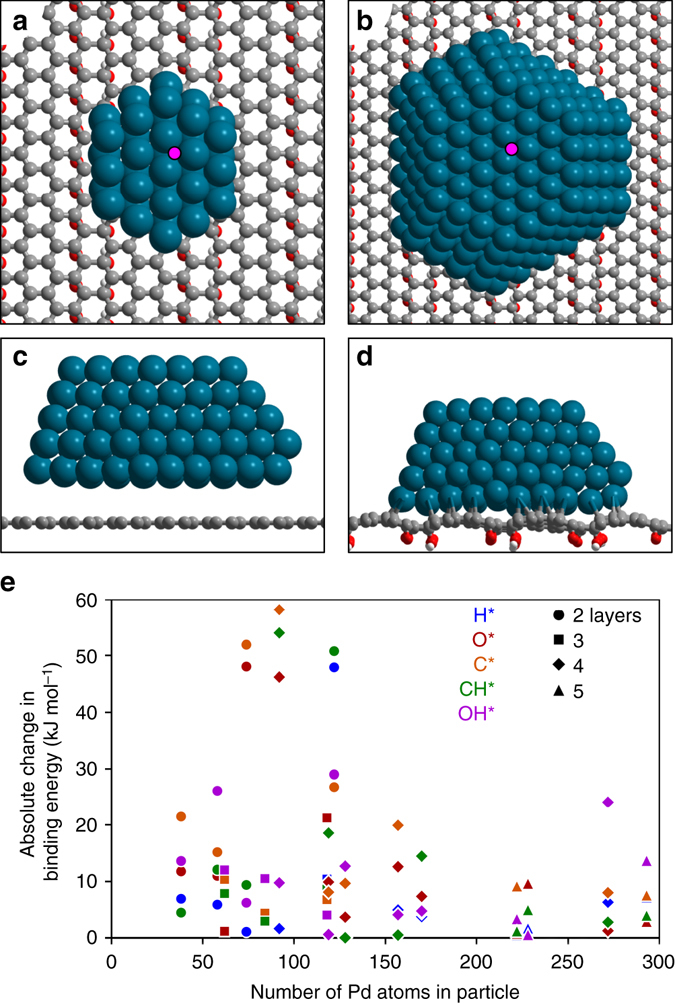
Theoretical calculations for Pd/C. **a** Pd_38_ and **b** Pd_293_ clusters bound to a functionalized graphene support; *pink dot* shows binding location of probe adsorbates. **c** Side-view of Pd_293_ cluster above graphene. **d** Side-view of Pd_293_ cluster bound to functionalized graphene support. **e** Absolute differences in binding energies of H* (*blue*), O* (*red*), C* (*orange*), CH* (*green*), and OH* (*purple*) on sites shown in *pink* in parts **a**, **b** on Pd particles ranging from 38 to 293 atoms and having 2 (circle), 3(square), 4(diamond) and 5(triangle) metal layers

Periodic DFT calculations were used here to elucidate the differences in metal-support interactions observed with defunctionalization (PS-PS800) and graphitization (PS, LHT, HHT). The interaction energies and binding geometries of sixteen Pd nanoclusters on carbon supports (graphitic or functionalized with oxygen and hydroxide) were examined. The Pd nanoclusters varied in size from 38 (Fig. 6a) to 293 atoms (Fig. 6b) and had 2–5 metal layers orthogonal to the basal plane of carbon. Interaction energies for Pd clusters on a pure graphene support were < 5 kJ mol^−1^ Pd^−1^_interface_, whereas those on oxidized graphene ranged from 30 to 50 kJ mol^−1^ Pd^−1^_interface_ (exothermic), indicating a significantly stronger interaction between the metal and the functionalized carbon support. These shifts in interaction energy were also observed in the binding geometries, as Pd clusters merely physisorb above graphene supports (as shown in Fig. 6c for a 293-atom Pd particle), while they chemisorb to the functionalized graphene (Fig. 6d for a 293-atom Pd particle), in agreement with the TEM images of the Pd–C interface (Fig. 5). These results corroborate the Raman (Supplementary Table 1) and C1s XPS (Supplementary Fig. 2) data and suggest very weak metal-support interactions for Pd/LHT and Pd/HHT.

The binding energies of the probe adsorbates H^*^, O^*^, C^*^, CH^*^, and OH^*^ at three-fold sites far from the Pd–support interface (shown with pink dots in Fig. 6a, b) were also shifted by changes in the underlying support, although the magnitude of their shift decreased as particles became larger (Fig. 6e). The largest particle (293 atoms) studied here using DFT is a rough hemisphere ~ 2.5 nm in diameter and ~ 1 nm in height, significantly smaller than the ~ 5 nm diameter particles catalytically tested. Smaller particles (~ 100 atoms) showed shifts in binding energies near 50 kJ mol^−1^, indicating major changes in the catalytic behavior of these sites. These results suggest that atoms near the Pd–support interface may be significantly altered and that the magnitude of these alterations may depend on the carbon’s surface chemistry, leading to changes in adsorption properties, electronic structure, and selectivity as shown in Fig. 3.

**Fig. 7:**
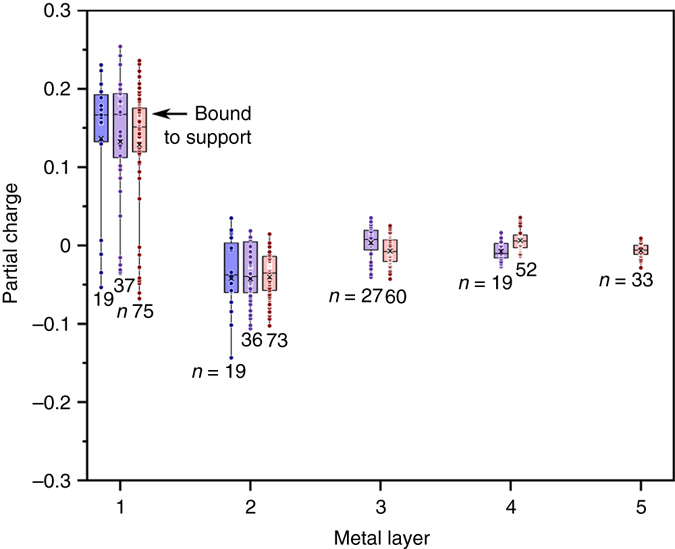
Partial charge analysis. Partial charges (QUAMBO) of each Pd atom, organized by metal layer for a 38-atom (*blue*, 2 layers), 119-atom (*purple*, 4 layers), and 293-atom (*red*, 5 layers)

QUAMBO charge analysis was performed on three particles of varying size (38, 119, and 293 atoms) bound to a functionalized graphene sheet as shown in Fig. 7. Pd atoms bound to the support have median partial charges of +0.15 to +0.17, independently on particle size. This charge transfer is consistent with the appearance of the Pd^δ+^ contribution in the XPS spectra. Specifically, we estimated the thickness of the Pd^δ+^ phase based on the Pd^0^/Pd^δ+^ atomic ratio determined from the XPS Pd 3d5/2 peaks (Supplementary Table 6) and assuming hemispherical particles of 5 nm in diameter (Fig. 5, Supplementary Fig. 8, Supplementary Table 5). These calculations show that the Pd^δ+^ contributions are consistent with a partial charge distributed over the first 1–2 atomic layers near the palladium–carbon interface (Supplementary Table 8), in good agreement with the QUAMBO charge analysis.

Metal atoms in the second layers of all three particles are consistently negatively charged through a compensation effect. This charge is much smaller, in the order of −0.04, and likely falls within the energy resolution of the XPS technique, making it experimentally undetectable. Metal atoms further from the support show little overall charge, indicating that these charge effects are localized and dissipate with distance from the surface, similar to the weaker shifts in binding energies observed with increasing particle size in Fig. 6e.

## Discussion

Carbon is typically considered an inert catalyst support compared to conventional oxides as it lacks Lewis acid sites and presents only weak Brønsted acid–base properties. Yet, there are numerous examples in the open and patent literatures showing that the activity and selectivity of carbon-supported catalysts are sensitive to the choice of the carbon scaffold^19–21, 31^. These support effects remain poorly understood and, in the case of cinnamaldehyde hydrogenation, they were attributed to differences in the reactant’s adsorption mode depending on surface polarity^20, 21, 36^. However, this interpretation underestimates the structural and chemical complexity of carbon supports and, in particular, the electronic effects that may take place at the metal–carbon interface. Recent works on single-atom catalysis suggest that heteroatoms decorating defect sites play a role similar to ligands in homogeneous catalysis, where the activity of metal centers can be modulated through electron donation and withdrawal^57^. Obviously, ligand effects as defined in the ligand field theory are spatially constrained as they require orbitals of the metal atom and its ligands to overlap. The impact of these electronic effects on metal nanoparticles of 5 nm size, which consist of hundreds of atoms, is negligible: assuming hemispherical nanoparticles with 11% of the Pd atoms at the interface and an oxygen-to-carbon ratio of 0.25, at most ~ 2.75% of the total Pd atoms would be altered through ligand effects. This small percentage of Pd atoms located at the interface and influenced by ligand effects is inconsistent with the Pd^0^/Pd^δ+^ ratios determined by XPS and the trends we observed (Fig. 3). Yet, the correlations we established between support properties and hydrogenation performance demonstrate that carbon scaffolds play a non-innocent role on the catalytic activity of large nanoparticles. The detailed characterization of our catalysts allowed us to rule out common artefacts related to variations in metal loading, particle size, particle shape, and mass transfer limitations. The reaction conditions we selected also allowed us to exclude contributions from hydrogen spillover and palladium β-hydride. Finally, correlations between catalytic activity, chemisorption, XPS, TEM, Raman spectroscopy, and the differences in their respective measurement conditions (different temperatures, pressures, and gas/vacuum environments) are consistent with a charge transfer from the metal to the carbon scaffold for Pd supported on the PS-PS800 SCCNT series. Our theoretical calculations also corroborate this interpretation. The QUAMBO charge analysis indicates strong EMSI that alter 20% of the Pd atoms, in agreement with the Pd^0^/Pd^δ+^ atomic ratio determined by XPS. While the partial charge seems to be only distributed on the metal atoms near the interface with the support, these effects dissipate over several layers, as indicated by the calculations performed with probe adsorbates (Fig. 6).

The correlations between Pd^δ+^ intensity and support surface functionalization can be explained by the changes in electronic properties of the support. The decrease in the work function (*Φ*) of carbon scaffolds with decreasing oxygen functionalities has been shown^58, 59^. Theoretical calculations have revealed that common oxygen functionalities (epoxy, carbonyl, hydroxyl) cause an increase of up to 60% of graphene’s computed work function due to the formation of C–O dipoles^59^. The shift in *Φ* was found to depend on oxygen concentration and the nature of the O-containing functional groups, hence the intensity of the corresponding dipole moment. For the present study, the decrease in oxygen concentration with annealing temperature (Supplementary Table 1) would progressively lower *Φ* of the graphenic layer in the studied SCCNTs, thus increase the difference between the *Φ* of Pd (5.2–5.6 eV) and the support. This difference has been reported to influence charge transfer in the case of metal oxide supports with EMSI effects^10, 11^. The interpretation is further supported by previous studies on cinnamaldehyde hydrogenation, which demonstrated that the selectivity towards C=C or C=O bond hydrogenation scales with the position of the d band center of the metal active phase. Metals with a d band center far from the Fermi level (e.g., Pt) are selective towards CALC, while Pd is more selective towards HCALD. This selectivity can be further modulated through ligands and promoters. For example, it was shown that the addition of Co, an electropositive element, provides electron density to Pt, hence lowers its d band center (more negative) and further enhances the selectivity to CALC due to the preferential formation of di-σ metal C–O bond^35^. Here, the increase in HCALD selectivity with Pd^δ+^, thus the electron depletion of the metal active phase and the up-shift of its d band center, is fully consistent with this interpretation.

The above analysis does not only correlate changes in selectivity with the electronic properties of the studied supports, but the results for the PS-PS800 series also pave the way for a quantitative control of these effects in order to achieve a desired selectivity. Specifically, this study shows that the catalytic activity of a desired metal can be controlled by selectively functionalizing or defunctionalizing (e.g. by annealing) the surface of the carbon support.

In summary, the influence of support surface functionalization and graphitization on electronic and adsorption properties of the active metal phase was elucidated in this work. The correlations between support functionalization, charge distribution at the metal–carbon interface, and catalytic selectivity were established. These correlations provide insights into how carbon support can be utilized for tuning the electronic properties of the metal decorating its surface through Schwab effects, hence its catalytic performance. The present work is expected to set the foundations of general rules that will enable the rational design of carbon-supported catalysts.

## Methods

### Catalyst synthesis

Stacked cup carbon nanotubes (SCCNTs) were obtained from Pyrograf Products, Inc (Ohio, USA). The SCCNTs were synthesized by chemical vapor deposition (CVD) and annealed at 700, 1500, 3000 °C post synthesis to obtain the pyrolytically stripped (PS), low heat treated (LHT), and high heat treated (HHT) supports, respectively. Detailed characterization of the SCCNTs can be found elsewhere^28^. In total 10 g of SCCNTs were functionalized using 500 ml of trace metal grade concentrated nitric acid at 100 °C for 4 h, followed by washing with 5 liters of deionized water and drying overnight at 110 °C. The PS SCCNTs were divided into separate batches and thermally defunctionalized in flowing nitrogen at 200, 400, 600, 800, 1000 °C for 30 min to obtain the PS200-PS1000 series of supports. The increased treatment temperature for the PS200-PS1000 series removes oxygen functionalities of the supports.

Pd was deposited on the supports in the form of nanoparticles using incipient wetness impregnation. Typically, 125 mg of palladium (II) nitrate dihydrate was dissolved in 8.4 ml of deionized water (at pH 4.0, adjusted using nitric acid) and the solution was added dropwise to 1 g of the supports, followed by sonication for 5 min. The samples were dried overnight at 65 °C followed by drying at 80 °C for 8 h in an oven. The impregnated and dried SCCNTs were then placed in a tube furnace and calcined in air (200 ml min^−1^), while heating at 2 °C min^−1^ to 250 °C and held at this temperature for 2 h. The calcined catalysts were cooled down to room temperature and were flushed with nitrogen gas for 30 min and subsequently reduced under hydrogen (200 ml min^−1^) at 400 °C for 2 h. The gas flow rates were controlled using Brooks mass flow controllers.

### Raman spectroscopy

The nitric acid-treated SCCNTs supports were characterized using Raman spectroscopy to estimate the ratio of graphitic to defective carbon of the supports. A Renishaw inVia Raman Microscope equipped with a coherent Argon ion laser was used for the analysis. Each sample was analyzed at three different spots to ensure homogeneity of the sample. Ten accumulations with a 30 s acquisition time, 25 mW power of a laser line at 488 nm were used for each analysis. The measurement range from 800 to 2000 cm^−1^ was used for analysis. The data analysis was conducted using the method described by Knauer et al.^60^ According to this method, a multi-component baseline fit and deconvolution was used: bands at 1580 cm^−1^ (G-band), 1350 cm^−1^ (D_1_-band), 1620 cm^−1^ (D_2_-band), and 1200 cm^−1^ (D_4_-band) were fit with Lorentzian peaks while the contribution at 1500 cm^−1^ (D_3_-band) was fit with a Gaussian peak (Supplementary Fig. 1). The Lorentzian peak at 1350 cm^−1^ (D_1_) is characteristic of the defective carbon atoms within a graphene layer and the peak at 1580 cm^−1^ (G) represents the in-plane stretching of the sp^2^ hybridized carbon in the honeycomb carbon structure. The ratio of the graphitic to the defective carbon bands was estimated using the G and D1 peaks (Supplementary Table 1). The peaks D2 and D4 were attributed to the non-graphitic basic structural units, while the D3 peak was attributed to the non-graphitic or molecular carbon content of the samples by Knauer et al. in their work^60^. Since these peaks are not representative of the graphitic core but were a part of the amorphous carbon layer present on the surface, they were not considered for calculating the graphitic character of the supports in the present work.

### Inductively coupled plasma optical emission spectroscopy

The bulk homogeneity of the metal loading on the carbon supports was confirmed by analyzing the samples using Inductively coupled plasma-optical emission spectroscopy (ICP-OES) with a Perkin Elmer Optima 8000 instrument. Before analysis, 30 mg of each catalyst was placed in different crucibles and treated in a muffle furnace at 1000 °C for 6 h to burn off the carbon supports. The residual contents of the crucibles were soaked overnight in 5 ml aqua regia (1:3 volume ratio of nitric acid:hydrochloric acid) for dissolving the Pd. The aqua regia with the dissolved Pd was then carefully transferred to a 50 ml volumetric flask and the total volume was made up to 50 ml using DI water. The standards for the calibration curve were prepared using a 10 vol% aqua regia in water solution and the Pd ICP standard (1000 p.p.m.). Each catalyst was analyzed in quadruplicates.

### Scanning electron microscopy

The catalysts were characterized at the micro- and nano- scale by studying the catalyst morphology using a FEI Quanta 250 instrument operated at 10 kV. The catalyst samples were placed in aluminum pans mounted on carbon stubs. A solution of sticking tape soaked in hexane was added on the catalyst samples and the sample was levelled off to decrease the surface roughness. The samples were imaged at different magnifications and in different regions using both the secondary electron and back-scattered electron detectors.

### Transmission electron microscopy

Transmission electron microscopy (TEM) analysis was conducted using a JEOL 2100 FEG S/TEM microscope operated at 200 kV equipped with a spherical aberration corrector on the probe forming lens. The samples were dispersed in ethanol and deposited on a holey carbon coated TEM grid. For scanning transmission electron micrsocopy (STEM) high-angular annular dark field (HAADF) analysis, a spot size of 0.13 nm, a current density of 140 pA, a camera focal length of 8 cm, corresponding to inner and outer diameters of the annular detector of about 73 and 194 mrad, were used.

Aberration-corrected TEM was conducted using an FEI Titan 80–300 operated at 300 kV equipped with a Gatan US1000 CCD camera. The samples were dispersed in ethanol and deposited on a holey carbon coated TEM grid.

### X-ray diffraction

X-ray diffraction (XRD) analysis was conducted using a Siemens D-500 instrument equipped with a copper X-ray tube, a diffracted beam monochromator, and a scintillation detector. The measurements were conducted using a gold holder and a medium resolution slit. The samples were scanned from 10 to 70° two-theta with a step size of 0.05° and a dwell time of 3 s per step. Jade software was used to analyze the data and the Scherrer equation was used to estimate the crystallite size.

### Hydrogen chemisorption

Hydrogen chemisorption adsorption isotherm studies were conducted using a Micromeritics ASAP 2020C instrument. In total 50 mg of the sample was loaded in a chemisorption tube and packed using quartz wool. The sample was reduced in-situ at 350 °C followed by long evacuation steps to remove the adsorbed hydrogen. Hydrogen was then re-introduced in the system at increasing pressures (2–450 mm Hg) under isothermal conditions (35 °C) for studying the first adsorption isotherm and the amount of total adsorbed hydrogen (H_T_) was calculated. A long evacuation step was then applied to remove the weakly or reversibly bound hydrogen and then a second adsorption isotherm was recorded to estimate the reversibly adsorbed hydrogen (H_R_). The size of the Pd nanoparticles was obtained using H_T_ and the ratio of the H_R_/H_I_ was calculated to compare the catalysts.

### X-ray photoelectron spectroscopy

Lab source XPS studies were conducted using a Kratos Amicus ESCA 3400 instrument. The anode used for the analysis was Mg (240 W) with the photon energy of K alpha 1 peak at 1253.688 eV and K alpha 2 peak at 1253.437 eV. The samples were loaded on an indium foil used here as an internal standard. A dwell time of 1 s, step size of 50 meV, and a pass energy of 150 eV were used. Five sweeps were recorded for each sample. Sputtered In foil and HOPG samples were used as standards for data analysis for Pd and C. Data analysis was conducted using the Fitt-win software.

Synchrotron radiation studies were conducted during single bunch mode at the ISISS beamline of the Fritz Haber Institute located at the BESSY II synchrotron facility in Berlin. The high-pressure setup consists mainly of a reaction cell attached to a set of differentially pumped electrostatic lenses and a differential-pumped analyzer (Phoibos 150 Plus, SPECS GmbH), as described elsewhere^61^. The spectra were collected in normal emission in vacuum with a probe size of  ≈ 150 µm × 80 µm. Sample contamination was checked by survey spectra at the beginning of each experiment. For XPS analysis, the photoelectron binding energy (BE) is referenced to the Fermi edge, and the spectra are normalized to the incident photon flux. The photoionization cross section was considered according to the calculations of Yeh and Lindau^62^. Background correction was performed by using a Shirley background^63^. The spectra were fitted following the Levenberg–Marquardt algorithm to minimize the χ^2^. Peak shapes were modeled by using asymmetric Doniach–Sunjic functions convoluted with Gaussian profiles^64^. The accuracy of the fitted peak positions is ≈ 0.05 eV.

### High-pressure fourier transform infrared spectroscopy

In-situ Fourier transform infrared (FTIR) studies were conducted to determine the interaction between hydrogen and the Pd/C catalysts using a Nicolet 6700 Thermo Scientific spectrometer. Approximately 0.7 mg of the catalyst powder was pressed onto a KBr pellet of 1 cm diameter and 2 mm thickness. The sample was then placed in a high-pressure high-temperature cell (P/N 5850c, Specac Ltd, UK) at the focal point of the spectrometer sample compartment. The samples (Pd catalysts supported on PS, LHT, HHT, PS200, PS600, and PS1000) were activated under 1 atm of hydrogen at 100 °C for at least for 1 h then cooled down to room temperature for the IR measurements. The IR spectra were recorded after each heating cycle. The resulting spectra were analyzed to monitor changes in adsorbate concentrations. The pressures used for the analyses were 15, 100, and 200 psi (1, 7, and 14 bar).

### Catalytic tests

The catalytic performance was probed for the liquid phase hydrogenation of cinnamaldehyde. In total 100 mg of Pd/C catalyst, 40 ml dioxane, and 365 mg decane (internal standard) were added to a 100 ml 3-neck flask with a stir-bar. The flask was connected to a condenser with water at 4 °C. The reaction mixture was magnetically stirred at 500 r.p.m., and heated using an oil bath kept at 80 °C using an IKA-RCT magnetic stir plate equipped with a PT1000 thermocouple. Nitrogen and hydrogen were provided to the solution using a gas dispersion tube equipped with a fine frit (10–20 µm porosity). The gas-flow rate was controlled using a Bronkhorst mass flow controller. A rubber septum was connected to the third neck of the flask and an 18 gauge, 6 inch needle was inserted in it for sampling. The top end of the needle was wrapped with parafilm to avoid any losses due to evaporation of dioxane. In a typical experiment, nitrogen was sparged through the flask at 20 ml min^−1^ for 20 min at room temperature to remove any air from the system. The gas flow was then switched to hydrogen at 20 ml min^−1^ and the temperature of the oil bath was raised to 80 °C and maintained at this temperature for 30 min. 5 g of trans-cinnamaldehyde dissolved in 10 ml dioxane was then added to the flask and this time was marked as the beginning of the reaction. Samples were withdrawn at specific time intervals using a glass syringe in pre-cooled glass vials and placed in the refrigerator immediately to quench the reaction.

The reaction samples were diluted 15 times with dioxane, filtered using 0.22 µm nylon filters, and analyzed using an Agilent 7890 A gas chromatograph (GC-FID) with an HP-5 column (30 m × 320 µm × 0.25 µm). An injection volume of 1 µl with a split ratio of 20:1 at 20 ml min^−1^ was used. The inlet heater temperature was maintained at 300 °C with a total flow of 24 ml min^−1^ and a septum purge flow of 3 ml min^−1^. A column flow of 1 ml min^−1^ was used. The oven initial temperature of 40 °C was held for 1 min followed by ramping to 180 °C at 10 °C min^−1^. The detector heater temperature was maintained at 300 °C.

The product selectivities were calculated using Eq. (1):

1 $${\rm Selectivity\,}( {{\% }} ) = \frac{{( {\rm Moles}\,{\rm of}\,{\rm product}\,{\rm produced}\,{\rm at}}\,{t} )}{{({\rm Moles}\,{\rm of}\,{\rm reactant}\,{\rm converted}\,{\rm at}}\,{t})} \times 100$$

where, the moles of the product produced and the moles of reactant converted were calculated for same time t for the reaction.

### Density functional theory calculations

Plane-wave density functional theory calculations were performed using the Vienna ab initio simulation package (VASP)^65^ to calculate interaction energies between Pd particles and C-supports (with and without functionalization) and binding energies of probe adsorbates (H^*^, O^*^, C^*^, OH^*^, and CH^*^). Plane-waves were constructed using an energy cutoff of 400 eV with projector augmented wave potentials^66^. The revised Perdew–Burke–Ernzerhof (RPBE) form of the generalized gradient approximation (GGA) was used to determine exchange and correlation energies^67, 68^. Wavefunctions were converged to within 10^−6^ eV and forces were computed using a fast Fourier transform grid with a cutoff of twice the planewave cutoff. A 1 × 1 × 1 Γ-point sampling of the first Brillouin zone (k-point mesh) was used and structures were relaxed until forces on unconstrained atoms were <0.05 eV Å^−1^. Converged wavefunctions were transformed into a set of localized quasiatomic orbitals (QUAMBOs)^69^ and used to carry out Löwdin population analyses^70^ to determine the charges on the individual atoms.

Pd particles were modeled as cubo-octahedral particles which are composed of (100) and (111) surfaces. The dimensions of these surfaces were varied to generate sixteen particles ranging from 38 to 293 Pd atoms arranged in 2–5 metal layer orthogonal to the C-support supported on a single graphene sheet.

The C-support was modeled as either a single-layer graphene sheet, a two-layer graphene sheet, or a functionalized graphene sheet (which has 0.125 ML of O^*^ and 0.125 ML of OH^*^ adsorbed). The lattice parameters for the functionalized graphene sheet were 8.702 and 2.512; those for an equivalent pure graphene sheet were 8.523 and 2.46, indicating that functionalization of the graphene sheet is associated with an expansion in C–C bonds. The functionalized graphene sheet, furthermore, is curved in a periodic manner, further increasing C–C bond lengths in a manner consistent with the rehybridization of C-atoms from sp^2^ to sp^3^.

Probe adsorbates H^*^, O^*^, C^*^, OH^*^, and CH^*^ were modeled on the Pd particles at sites far from the Pd–C interface (at the center-most three-fold site on the (111) surface opposite the Pd–support interface). Binding energies for these adsorbates were compared when the support was pure graphene and when the support was functionalized graphene using Eq. (2):

2 $$\Delta {\rm{BE}} = \left| E[ {{\rm adsorbate}}\;{{\rm on}}\;{{\rm Pd}}\;{{\rm on}}\;{{\rm functionalized}}\;{{\rm graphene}}] \right. \hskip3pc\\ - E[ {{\rm Pd}}\;{{\rm on}}\;{{\rm functionalized}}\;{{\rm graphene}} ]  \hskip7.4pc\\  \left.- \left( E\left[ {{\rm adsorbate}}\;{{\rm on}}\;{{\rm Pd}}\;{{\rm on}}\;{{\rm graphene}} \right] - E\left[ {{\rm Pd}}\;{{\rm on}}\;{{\rm graphene}} \right] \right) \right|$$

Where ΔBE represents the absolute value of the shift in binding energy of the probe adsorbate.

### Data availability

The data that support the findings of this study are available from the corresponding author upon reasonable request.

## Electronic supplementary material

Supplementary Information
