# Supplementary material for: Interfacial charge distributions in carbon-supported palladium catalysts
Source: Nat Commun. 2017 Aug 24;8:340. doi: 10.1038/s41467-017-00421-x (PMC5569089; doi:10.1038/s41467-017-00421-x)
Supplement: Supplementary file 1 — Supplementary Information [file 41467_2017_421_MOESM1_ESM.pdf]

Description of Supplementary Files

File Name: Supplementary Information
Description: Supplementary Figures, Supplementary Tables and Supplementary References

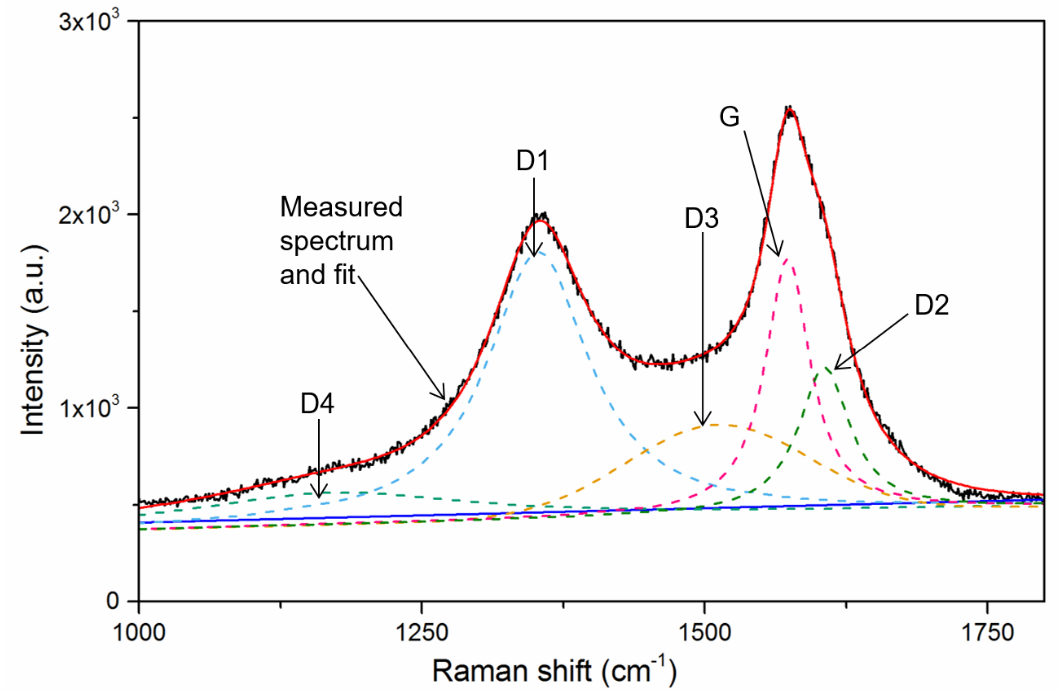

Supplementary Figure 1. Raman spectra analysis to determine the graphitic character of carbon supports. Raman spectrum obtained for the PS support post nitric acid treatment. Peak fitting was conducted using the method developed by Knauer et. al. consisting of 5 peaks—D1: disordered graphitic lattice carbon; D2: non-graphitic basic structural unit vibrations; D3: non-graphitic or molecular carbon; D4: non-graphitic basic structural unit vibrations; G: ideal graphitic lattice.[1]

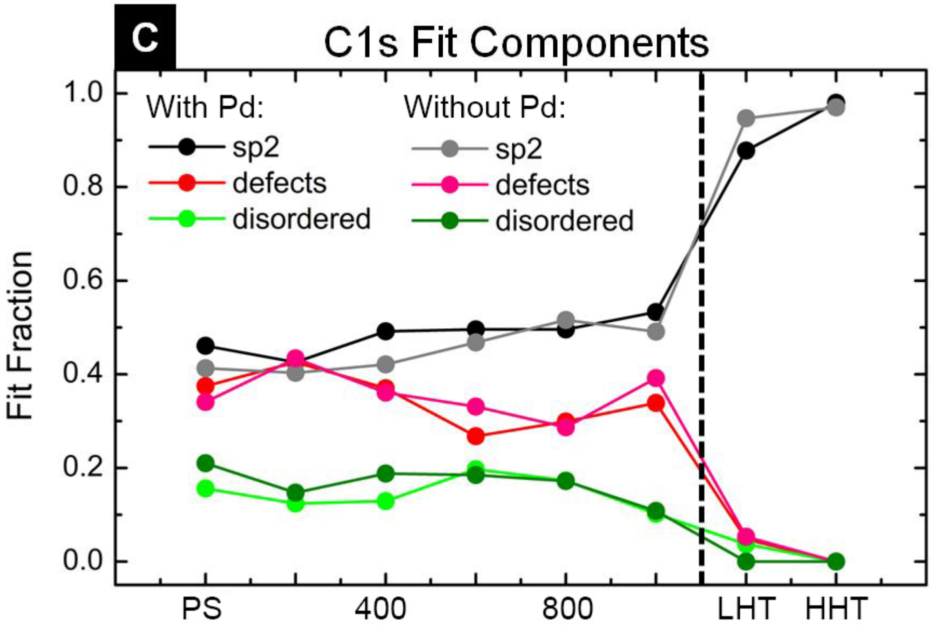

Supplementary Figure 2. Defect and graphitic character analysis of carbon supports using XPS. Evolution of the C1s fit components corresponding to sp^2 carbon, defective carbon (point-like defects), and disordered carbon (at edges and large vacancies) with annealing temperature. The data is based on deconvolved C1s spectra of the carbon supports with and without Pd acquired using synchrotron radiation for higher energy resolution and surface sensitivity. The fit fractions are normalized to the sum of all three fit components shown here.

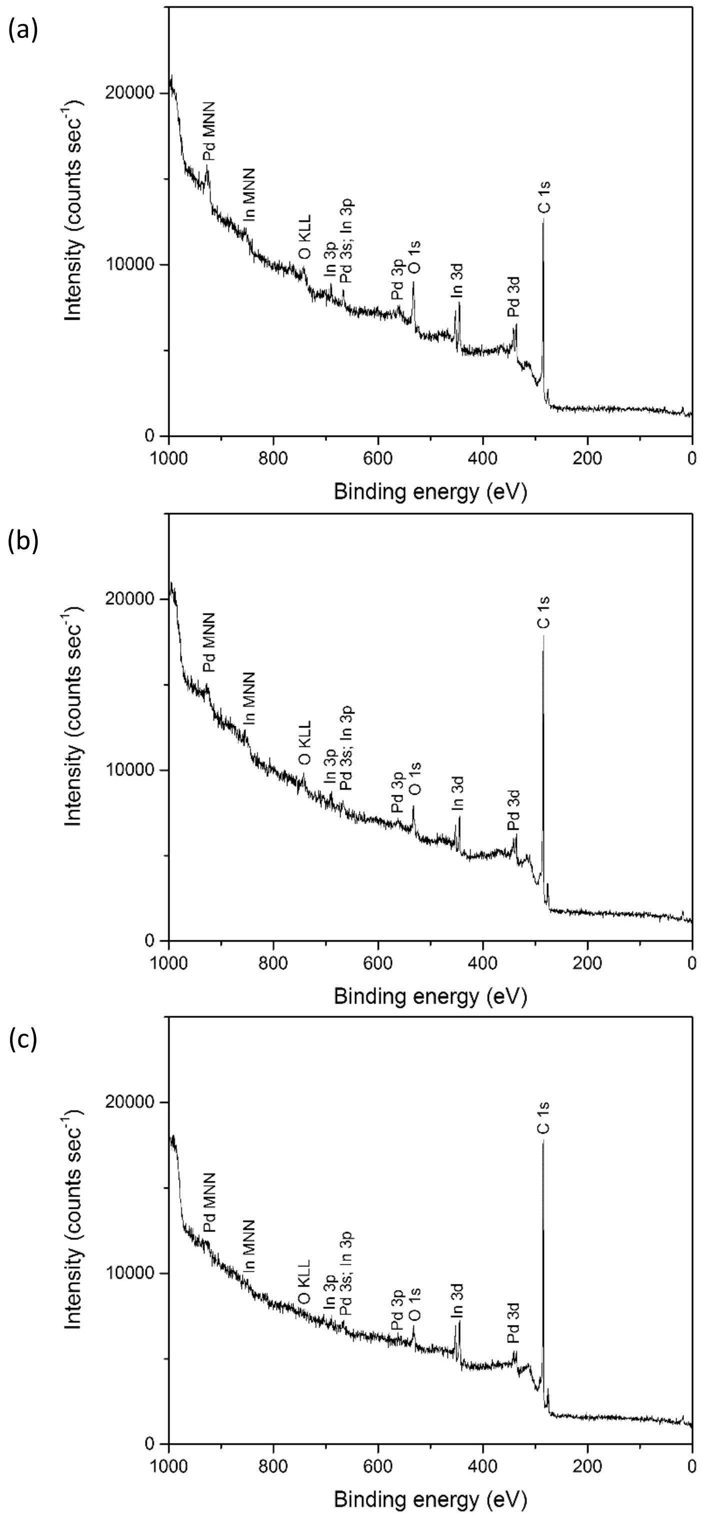

Supplementary Figure 3. XPS survey spectra of the supports after treatment with nitric acid. The detected signals correspond to C, O, Pd, and In (used as sample holder and internal standard) for the (a) PS, (b) LHT, and (c) HHT supports. The spectra confirm that the nitric acid treatment removed any inorganic impurities resulting from the synthesis and/or graphitization of the carbons supports.

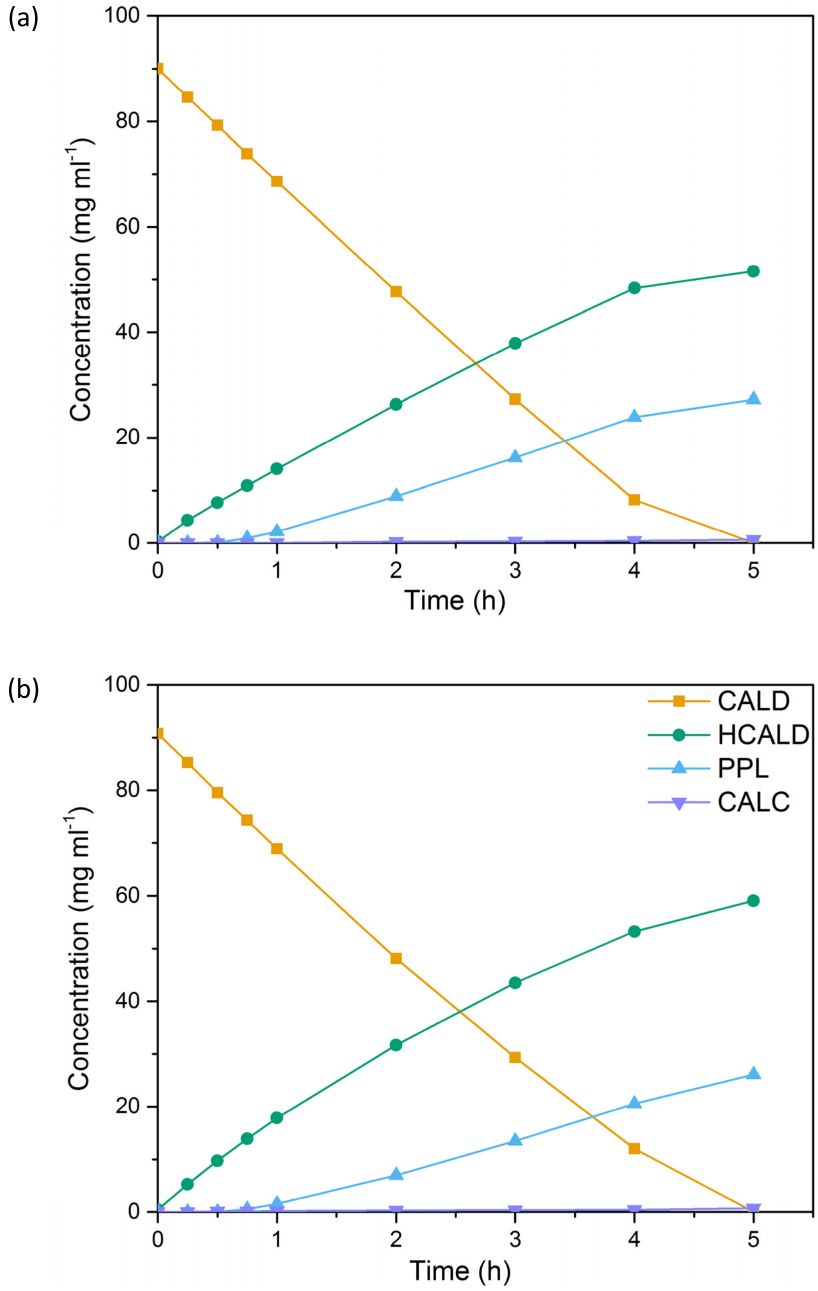

Supplementary Figure 4. Catalytic reaction profiles for CALD hydrogenation. Catalytic traces for (a) Pd/PS and (b) Pd/HHT catalysts for the batch reaction at 80 °C, hydrogen flowrate = 20 ml min^{-1}, stirring rate = 500 rpm, mass of catalyst = 100 mg, mass of cinnamaldehyde = 5 g.

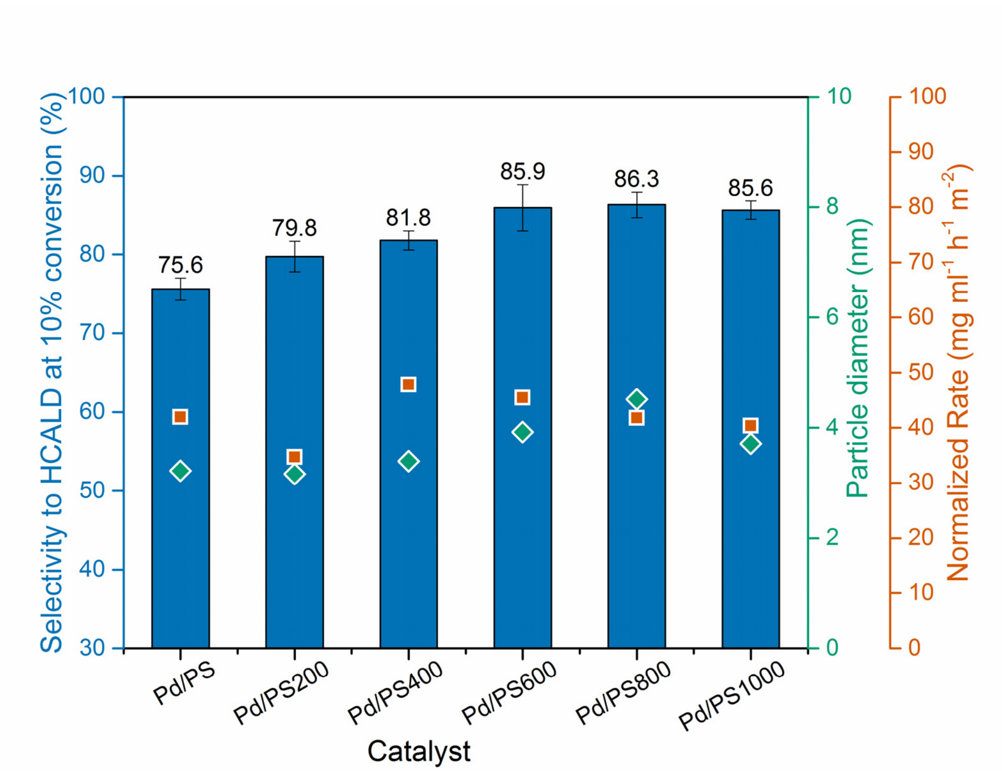

Supplementary Figure 5. Trends in HCALD selectivity for Pd supported on PS-PS1000. Selectivity to HCALD at 10% conversion of CALD for Pd supported on PS support and on PS defunctionalized at 200-1000 °C (blue). The values correspond to the average selectivity calculated for three independent experiments ± the corresponding standard deviation (s.d.). Comparing the selectivity results with Pd particle size (green) and normalized rate for CALD conversion (orange) shows no clear trend, indicating that the observed differences in selectivity are not due to obvious artefacts such as particle size effects.

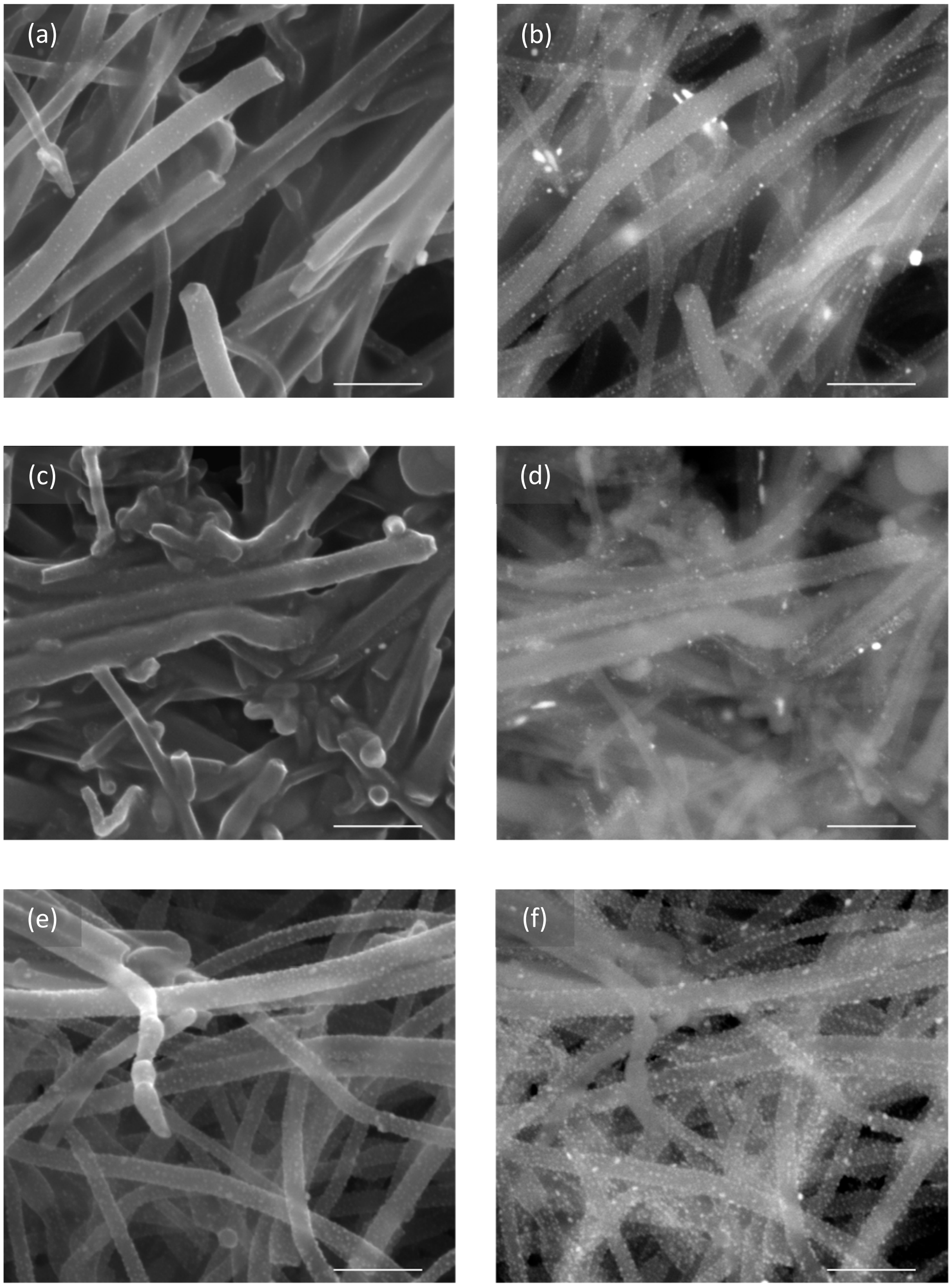

Supplementary Figure 6. Distribution of palladium nanoparticles on carbon supports. Scanning electron microscopy (SEM) images of the (a,b) Pd/PS400, (c,d) Pd/PS600, (e,f) Pd/PS800 showing the Pd dispersions typically achieved for the studied catalysts. Each region was imaged with both the secondary electron (SE) and back scattered electron (BSE) detectors for higher Z-contrast. A few large Pd particles were occasionally observed in these samples in BSE mode due to the higher sensitivity to heavy elements of this detector. Because of their low number and surface-to-volume ratio, these particles play an insignificant role in the present work. Scale bars in the images represent 500 nm.

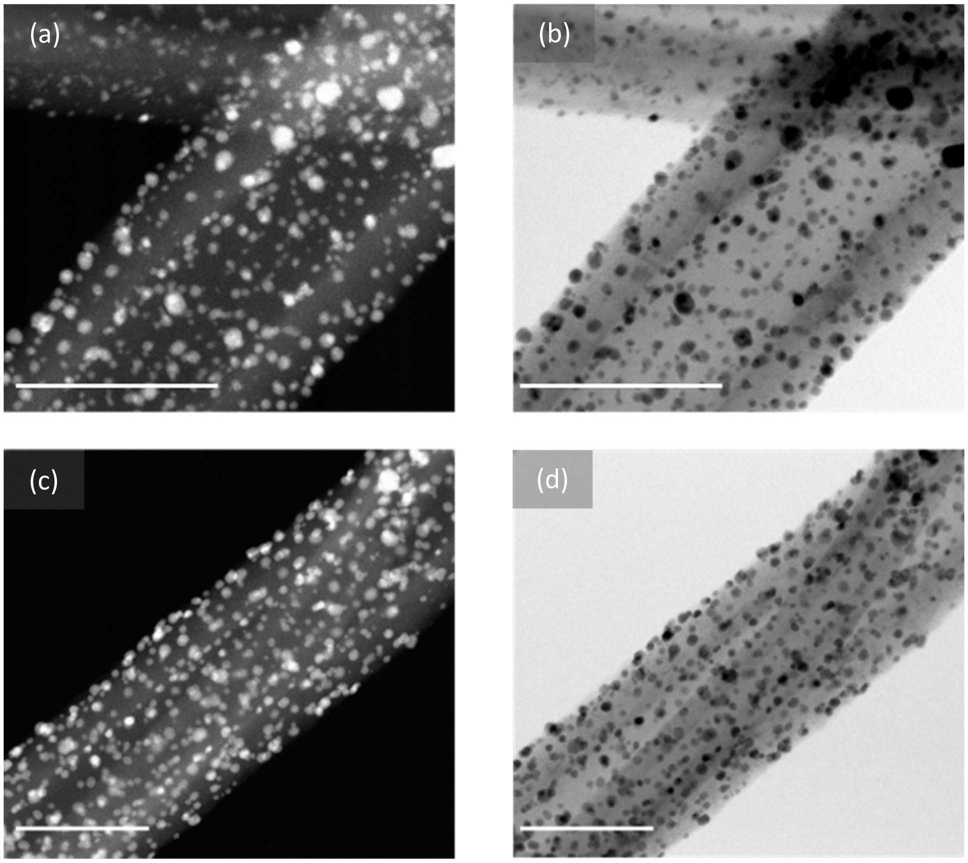

Supplementary Figure 7. Representative transmission electron microscopy micrographs of Pd/SCCNT catalysts. High resolution transmission electron microscopy (HRTEM) micrographs of (a,b) Pd/PS and (c,d) Pd/LHT acquired in high-angle annular dark-field (HAADF)-scanning transmission electron microscopy (STEM) mode (a,c) and bright field (BF)-STEM mode (b,d). Scale bars in the images represent 100 nm.

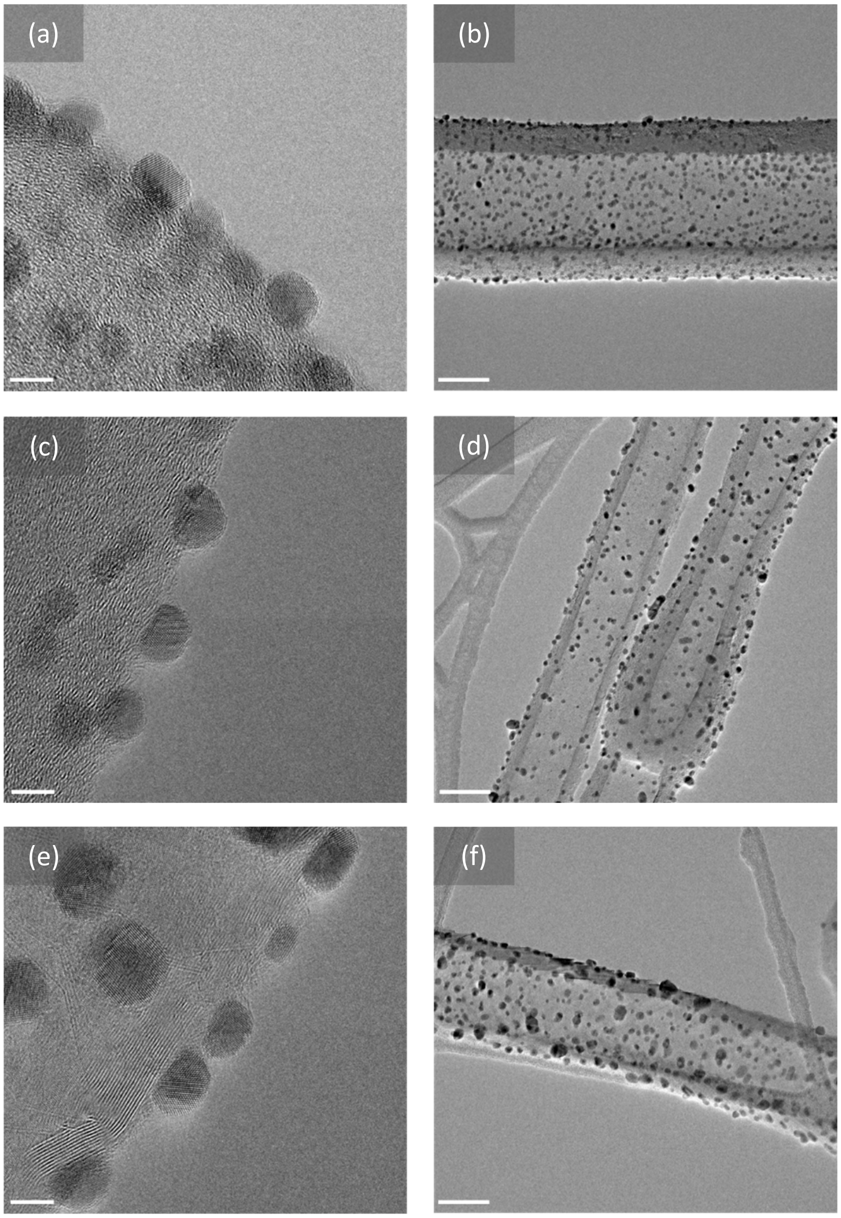

Supplementary Figure 8. Representative transmission electron microscopy micrographs of supported Pd nanoparticles. HRTEM micrographs of Pd nanoparticles supported on the (a,b) PS, (c,d) LHT, (e,f) HHT supports. Most Pd nanoparticles were hemispherical. Scale bars in the images represent 5 nm (a, c, and e) and 50 nm (b, d, and f).

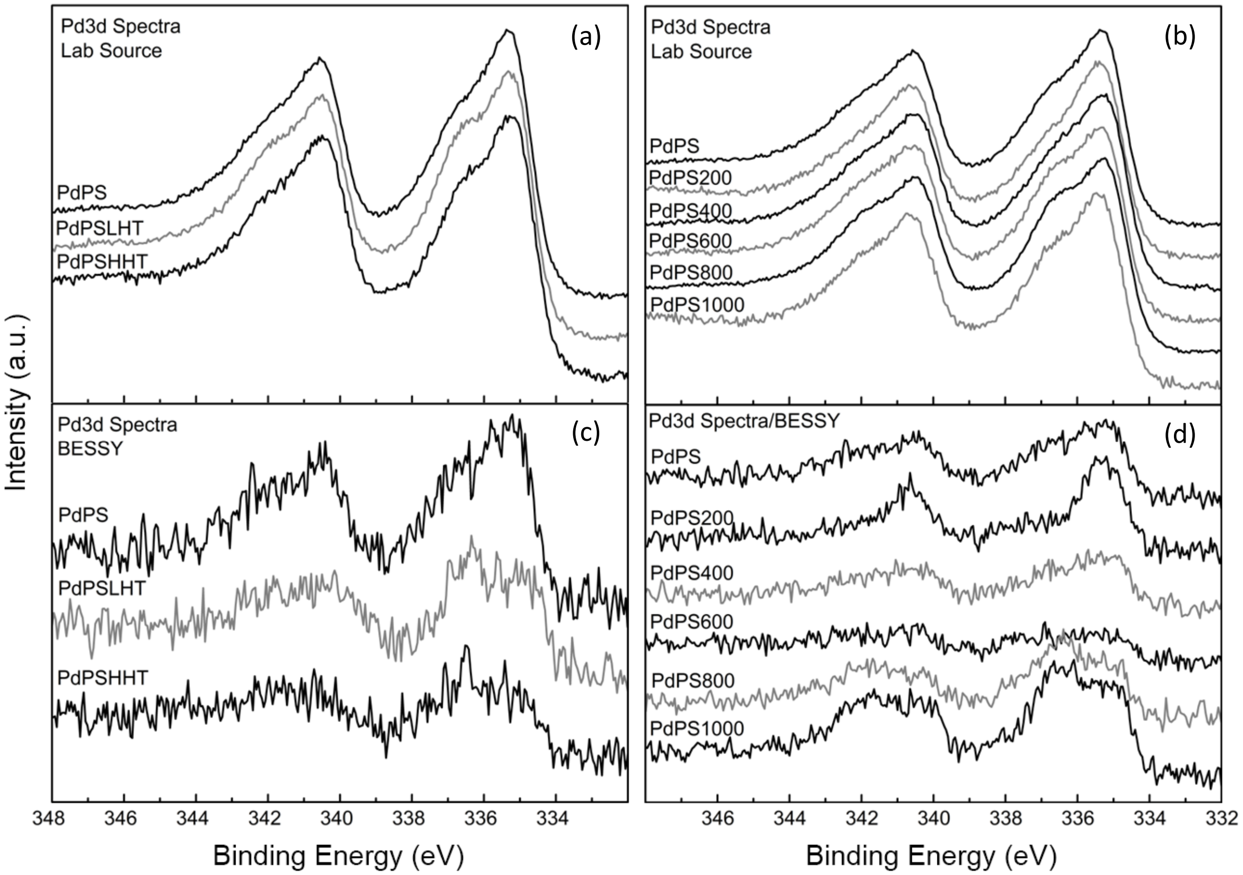

Supplementary Figure 9. Lab source and synchrotron radiation based X-ray photoelectron spectra. X-ray photoelectron spectra of Pd 3d using lab source (a, b) and synchrotron radiation (c, d). The spectra correspond to the Pd/PS-HHT series (a, c) and the Pd/PS200-PS1000 series (b, d). Note that single bunch operation uses a significantly lower ring fillings resulting in a beam current of ≤ 20 mA, which accounts for the relatively low signal-to-noise ratio for the synchrotron experiments.

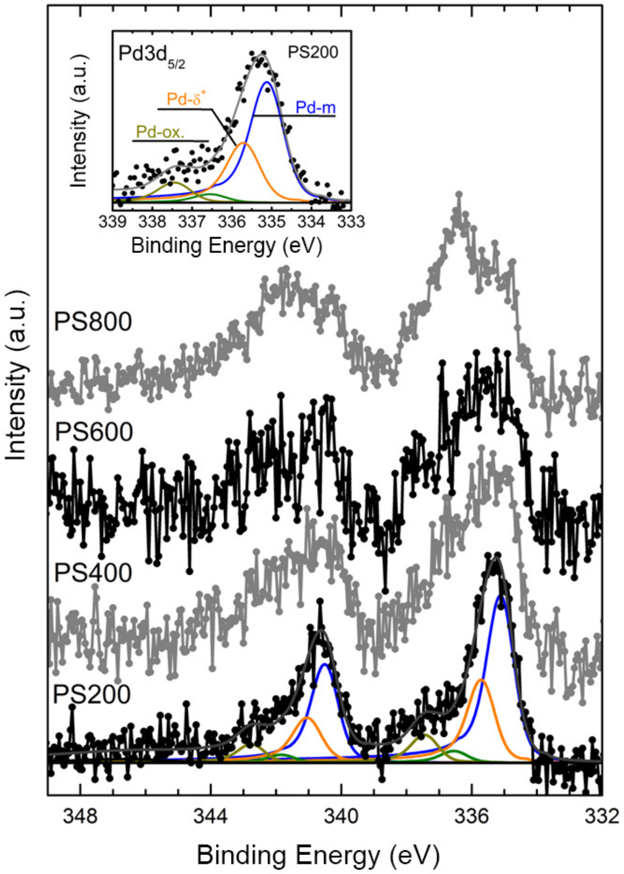

Supplementary Figure 10. Synchrotron based X-ray photoelectron spectra. X-ray photoelectron spectra of Pd/PS200 – Pd/PS800 catalysts using synchrotron radiation. The peak deconvolution for the Pd 3d 5/2 and 3/2 demonstrate the presence of Pd metal (blue), PdO and PdO_2 (green), and $Pd^{\delta+}$ (orange). Note that single bunch operation uses a significantly lower ring fillings resulting in a beam current of ≤ 20mA which accounts for the relatively low signal-to-noise ratio.

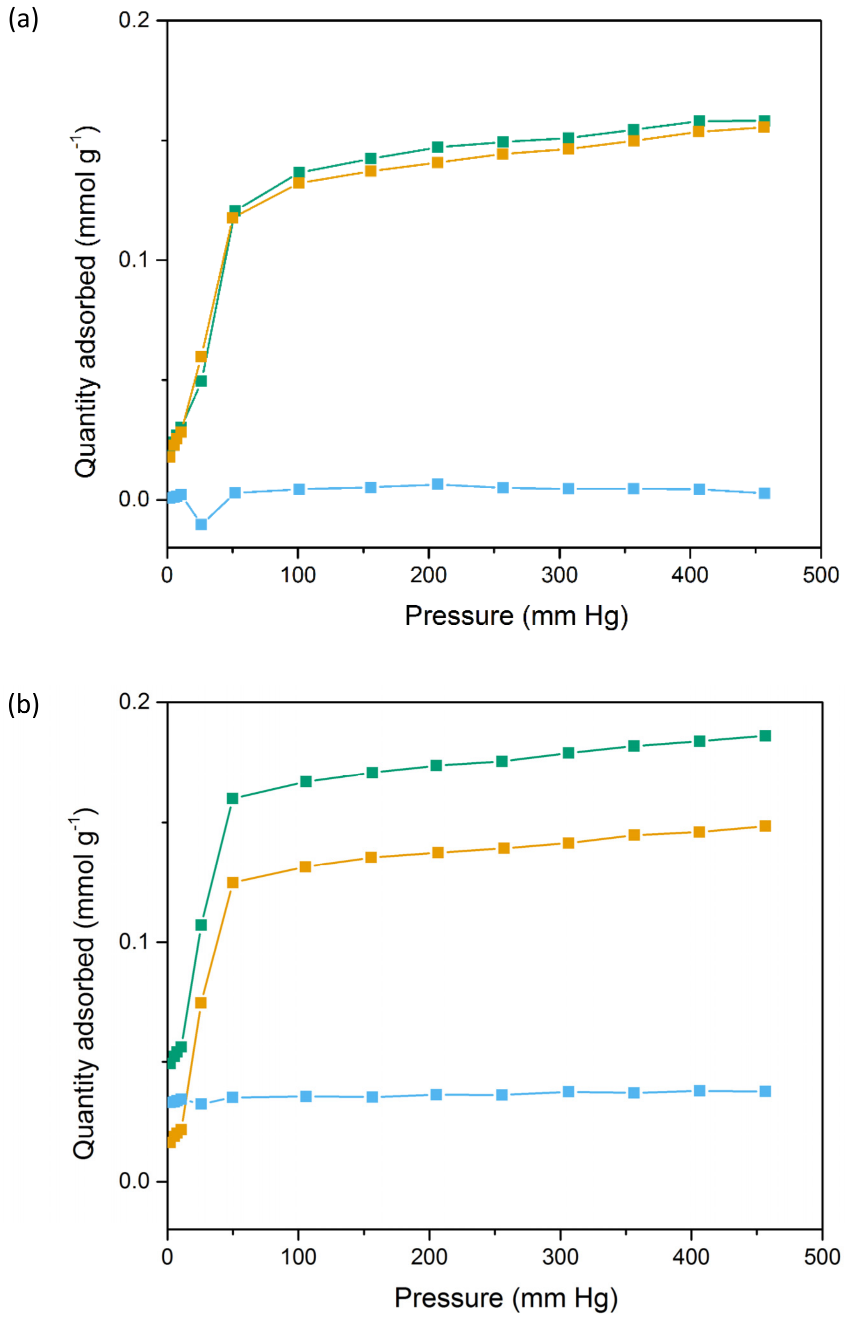

Supplementary Figure 11. Support effects on hydrogen adsorption isotherms. Hydrogen adsorption isotherms for (a) Pd/PS and (b) Pd/HHT catalysts. The graphs show the 1st adsorption isotherm (green), 2nd adsorption isotherm (orange), and the difference results (blue). Differences in reversibility can be observed for the Pd/PS and Pd/HHT catalysts. The ratios of the reversibly to irreversibly adsorbed hydrogen are given in the Supplementary Figure 12.

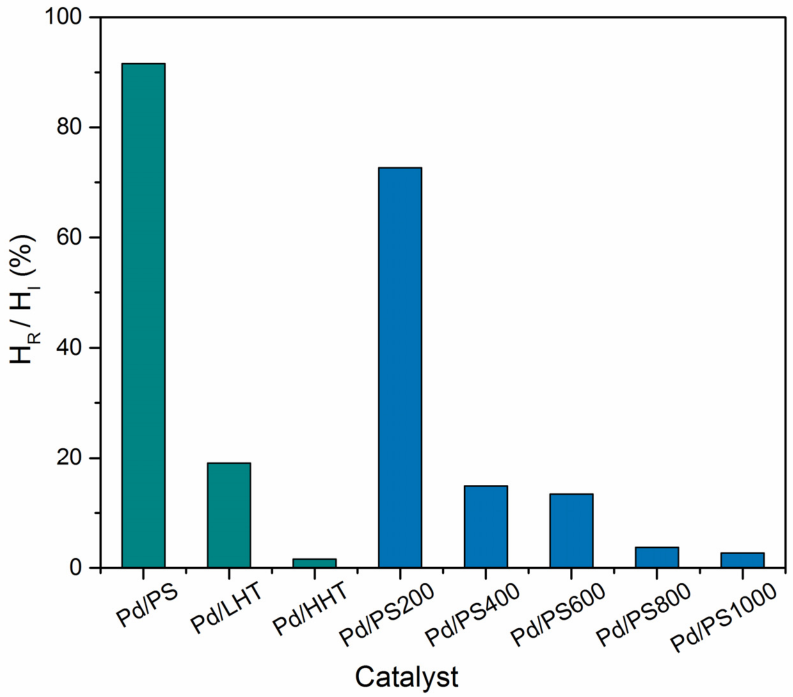

Supplementary Figure 12. Differences in hydrogen adsorption properties for the studied catalysts. Ratios of reversibly (H_R) and irreversibly adsorbed hydrogen (H_I) for the synthesized Pd/C catalysts.

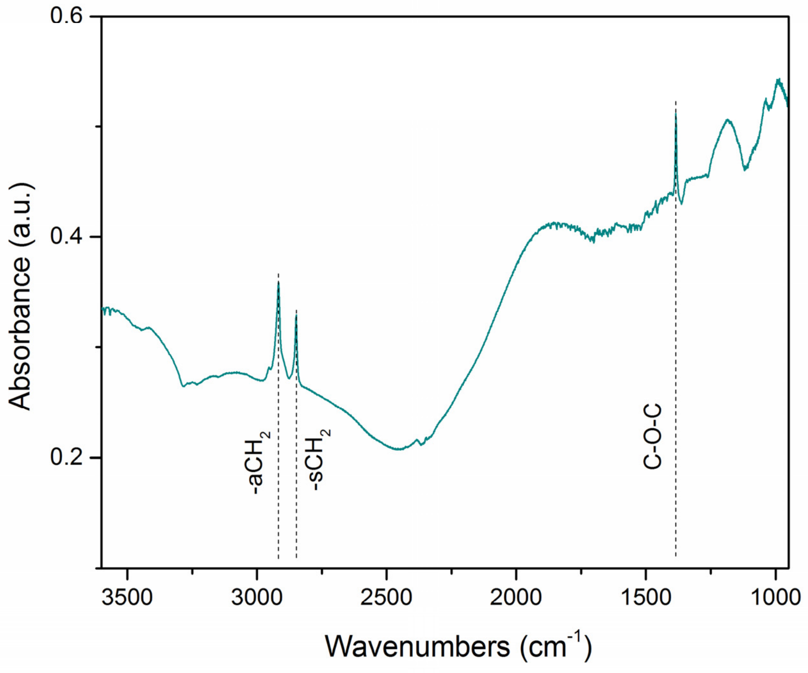

Supplementary Figure 13. Representative infrared spectrum of Pd/SCCNT in the absence of H_2. Infrared (IR) spectrum of the Pd/PS catalyst, recorded prior to H_2 exposure at room temperature, and referenced to the blank KBr pellet. The $–CH_2$ symmetric and asymmetric modes are observed at 2844 cm^{-1} and 2916 cm^{-1} and the C-O-C mode at 1385 cm^{-1}. The sample was activated under 1 bar of hydrogen at 100 °C for 1 h, then cooled down to room temperature for the IR measurements.

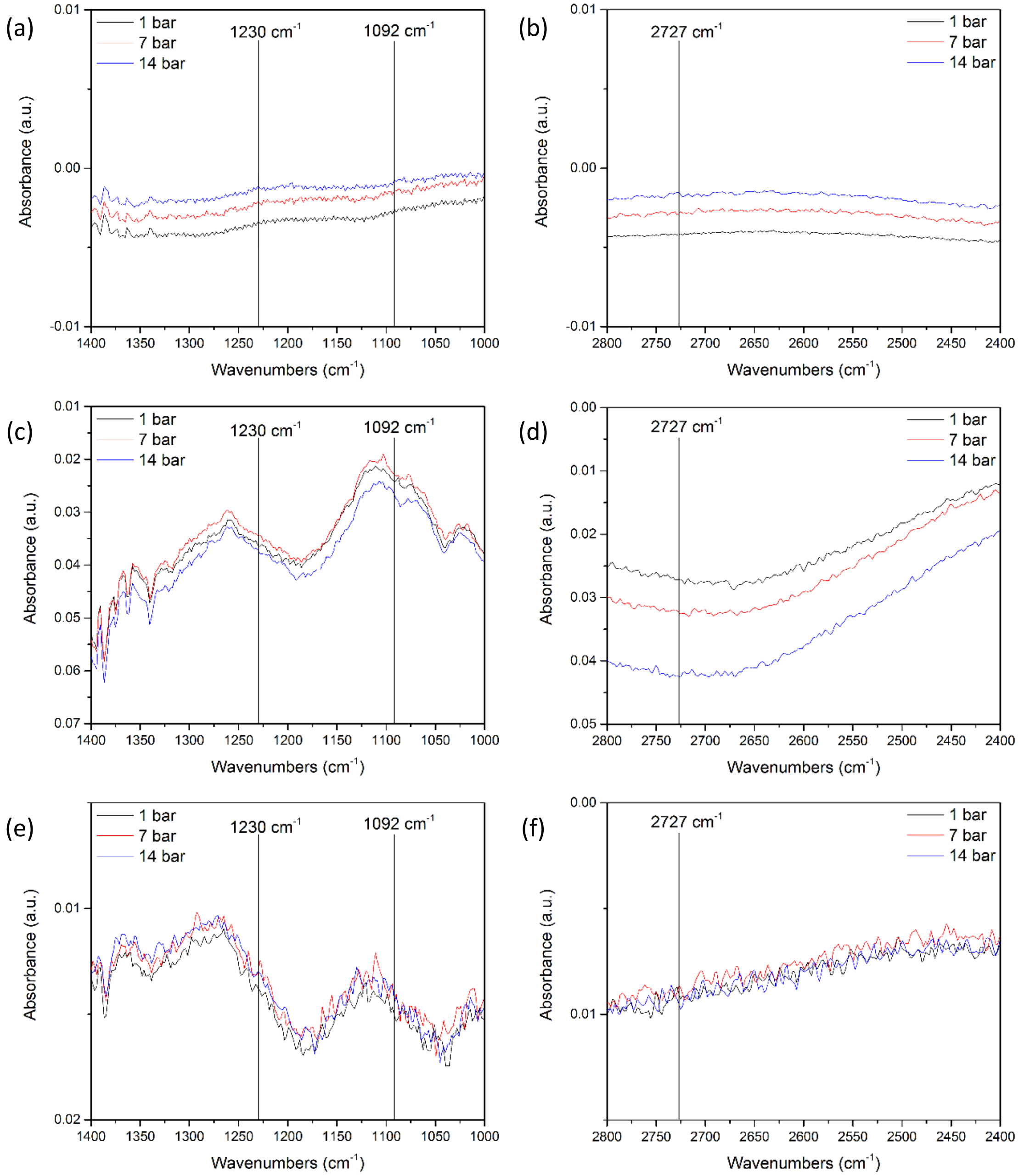
(a)
(b)
(c)
(d)
(e)
(f)
1 bar
7 bar
14 bar
1230 cm-1
1092 cm-1
2727 cm-1
Absorbance (a.u.)
Wavenumbers (cm-1)

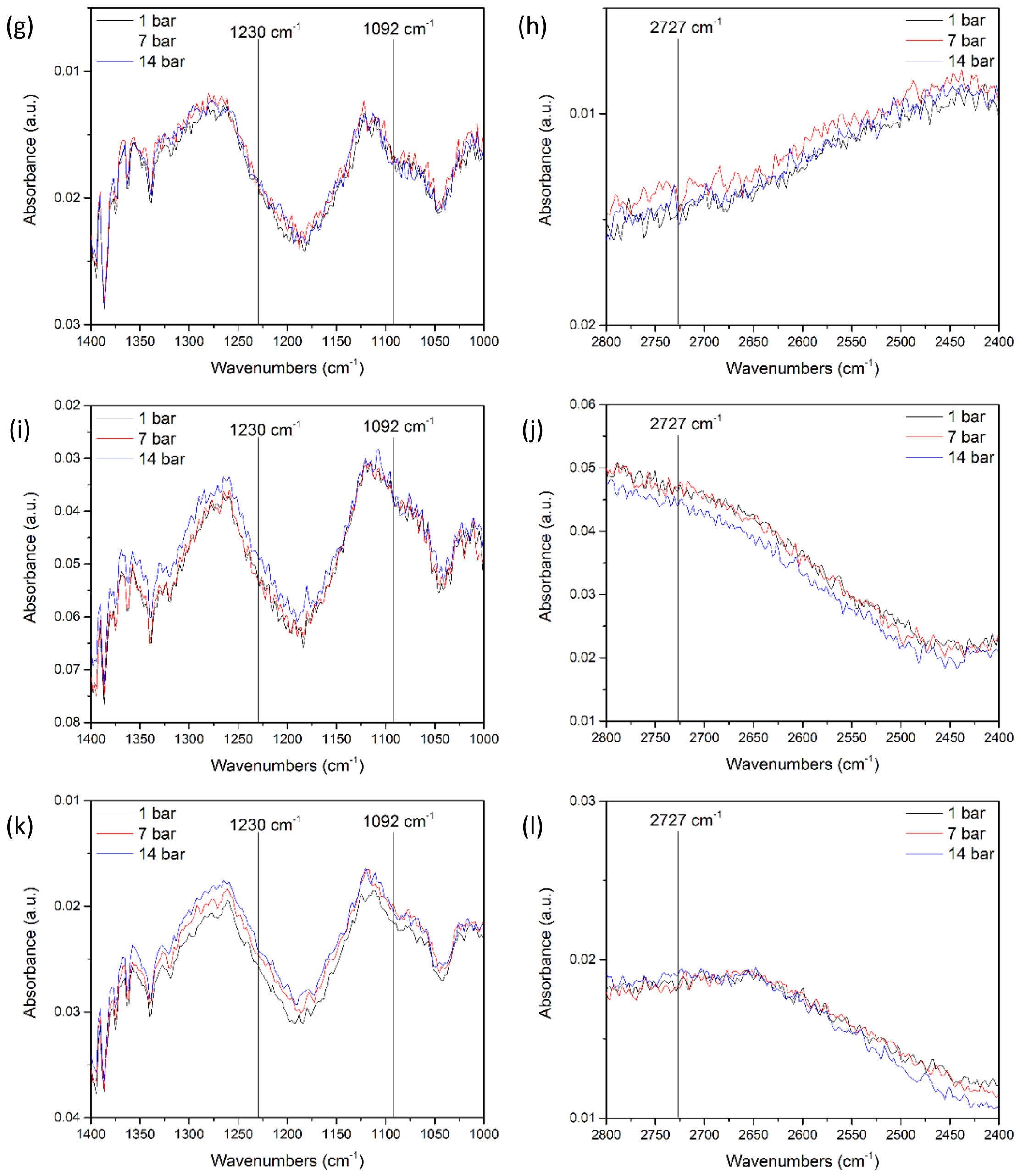

Supplementary Figure 14. High-pressure infrared spectra (HP-FTIR) of Pd/SCCNT in the presence of H_2. IR spectra of (a, b) Pd/PS, (c, d) Pd/LHT, (e, f) Pd/HHT, (g, h) Pd/PS200, (i, j) Pd/PS600, (k, l) Pd/PS1000 catalyst recorded under 1 (black), 7 (red), and 14 (blue) bar of hydrogen gas and subtracted from the corresponding spectrum recorded before exposure to H_2 at room temperature. Signals characteristic of the C-H modes at 2727 cm^{-1}, 1230 cm^{-1}, and 1092 cm^{-1} are observed at 14 bar only. The signal-to-noise ratios for all modes are too low to determine if there is any hydrogen spillover at 7 bar. Prior to these measurements, the samples were activated under 1 bar of hydrogen at 100 °C for 1 h, then cooled down to room temperature for IR measurements.

Supplementary Table 1. Effect of thermal treatments on the supports' graphitic character and functionalization. Raman G-band/D1-band intensity ratios for the studied carbon supports and corresponding O1s/C1s ratios calculated from the highest intensity of the XPS spectra acquired using synchrotron radiation (BESSY II). These Raman and XPS results show the impact of annealing on the graphitic character and surface chemistry of the supports.

Support	IG/ID[a]	O1s/C1s
PS	0.43	0.24
LHT	1.25	0.06
HHT	5.83	0.03
PS200	0.38	0.23
PS400	0.40	0.23
PS600	0.51	0.11
PS800	0.62	0.15
PS1000	0.62	0.09

[a] IG and ID represent the intensity of the G-band and D1 band respectively.

Supplementary Table 2. Catalytic performance of the synthesized Pd/SCCNT samples at 10% conversion. Hydrocinnamaldehyde (HCALD) selectivity, yield, and carbon balance at 10% cinnamaldehyde (CALD) conversion. The values correspond to the average calculated for three independent experiments ± the corresponding standard deviation (s.d.).

Catalyst	HCALD Selectivity (%)	HCALD Yield (%)	Carbon balance (%)
Pd/PS	75.6 ± 1.4	7.6 ± 0.1	99.4 ± 0.1
Pd/LHT	83.5 ± 2.7	8.3 ± 0.3	99.5 ± 0.9
Pd/HHT	83.2 ± 2.8	8.3 ± 0.3	99.7 ± 0.4
Pd/PS200	79.8 ± 1.9	8.0 ± 0.2	99.6 ± 0.1
Pd/PS400	81.8 ± 1.2	8.2 ± 0.2	99.7 ± 0.1
Pd/PS600	85.9 ± 3.0	8.6 ± 0.3	99.9 ± 0.2
Pd/PS800	86.3 ± 1.6	8.6 ± 0.2	99.8 ± 0.1
Pd/PS1000	85.6 ± 1.2	8.6 ± 0.1	99.6 ± 0.1

Supplementary Table 3. Catalytic performance of the synthesized Pd/SCCNT samples at 100% conversion. Hydrocinnamaldehyde (HCALD) selectivity, yield, and carbon balance at 100% cinnamaldehyde (CALD) conversion.

Catalyst	HCALD Selectivity (%)	HCALD Yield (%)	Carbon balance (%) [a]
Pd/PS	58.4	58.4	96.5
Pd/LHT	73.0	73.0	101.8
Pd/HHT	67.4	67.4	101.0
Pd/PS200	65.4	65.4	97.5
Pd/PS400	66.4	66.4	105.2
Pd/PS600	71.6	71.6	99.8
Pd/PS800	72.4	72.4	106.7
Pd/PS1000	75.7	75.7	103.3

[a] Carbon balance values calculated by accounting for the decrease in volume and moles due to sampling.

Supplementary Table 4. Palladium metal loading on carbon supports. Pd metal loadings determined by inductively coupled plasma-optical emission spectroscopy (ICP-OES).

Catalyst	Pd metal loading (%)[a]
Pd/PS	3.3 ± 0.3
Pd/LHT	4.2 ± 0.6
Pd/HHT	4.7 ± 0.2
Pd/PS200	3.2 ± 0.3
Pd/PS400	3.3 ± 0.1
Pd/PS600	3.9 ± 0.2
Pd/PS800	4.6 ± 0.5
Pd/PS1000	3.9 ± 0.3

[a] All samples were analysed in quadruplicates to obtain the standard deviation.

Supplementary Table 5. Palladium nanoparticles size. Average particle sizes obtained from hydrogen chemisorption adsorption isotherms, aberration corrected transmission electron microscopy (TEM), and X-ray diffraction (XRD).

Catalyst	Hydrogen chemisorption (nm)	TEM (nm)	XRD (nm)
Pd/PS	4.86	5.6 ± 1.3	4.2 ± 0.3
Pd/LHT	5.04	8.2 ± 2.5	6.7 ± 0.3
Pd/HHT	2.99	6.2 ± 2.2	7.7 ± 0.4
Pd/PS200	4.94	-	5.8 ± 0.4
Pd/PS400	5.08	-	6.9 ± 0.4
Pd/PS600	4.98	-	9.9 ± 0.6
Pd/PS800	4.89	-	7.8 ± 0.6
Pd/PS1000	4.81	-	6.7 ± 0.5

Supplementary Table 6. Analysis of the charge transfer between the metal and carbon support. Ratio of the $Pd^0/Pd^{\delta+}$ phase as observed from synchrotron based X-ray photoelectron spectroscopy (XPS) Pd 3d 5/2 peak components normalized to highest intensity.

Catalyst	$Pd^0/Pd^{\delta+}$
Pd/PS	2.29
Pd/LHT	2.75
Pd/HHT	2.43
Pd/PS200	2.00
Pd/PS400	1.83
Pd/PS600	1.62
Pd/PS800	1.54
Pd/PS1000	2.00

Supplementary Table 7. Hydrogen chemisorption adsorption isotherms. Hydrogen chemisorption results: amount of hydrogen adsorbed for the 1st and 2nd isotherm, difference results for Pd/C catalysts at 400 mm Hg hydrogen pressure, amount of reversibly adsorbed hydrogen, and ratio of reversibly to irreversibly adsorbed hydrogen.

Catalyst	1st adsorption isotherm- amount of hydrogen adsorbed (mmol g^{-1})	2nd adsorption isotherm- amount of hydrogen adsorbed (mmol g^{-1})	Difference results (mmol g^{-1})	Hreversible (mmol g^{-1})	Hreversible/ Hirreversible
Pd/PS	0.15812	0.15364	0.00448	0.00058 ± 0.00147	91.7
Pd/LHT	0.15603	0.14138	0.01465	0.00261 ± 0.00173	19.0
Pd/HHT	0.1839	0.14601	0.03789	0.03372 ± 0.00028	1.6
Pd/PS200	0.1602	0.15352	0.00668	0.00072 ± 0.00138	72.7
Pd/PS400	0.1529	0.14605	0.00685	0.00325 ± 0.00162	14.9
Pd/PS600	0.16112	0.14593	0.01519	0.00367 ± 0.00140	13.4
Pd/PS800	0.15294	0.13731	0.01563	0.01130 ± 0.00081	3.8
Pd/PS1000	0.13679	0.12029	0.0165	0.01449 ± 0.00083	2.8

Supplementary Table 8. Number of $Pd^{\delta+}$ atomic layers based on XPS. The ratio of the $Pd^0/Pd^{\delta+}$ phase as observed from synchrotron based X-ray photoelectron spectroscopy (XPS) Pd 3d 5/2 peak components (Supplementary Table 6) was used to estimate the number of atomic layers impacted by the $Pd^{\delta+}$ phase in Pd nanoparticles. The values were calculated based on the following assumptions:

(a) the Pd nanoparticles are hemispherical and have a diameter of 5 nm, which corresponds to ~9 atomic layers (Supplementary Table 5)
(b) the particles are homogeneously covered with 2 atomic layers (~0.5 nm) of surface oxides
(c) the penetration depth of XPS under these conditions is ~2 nm
(d) all the Pd atoms at the interface are in the $Pd^{\delta+}$ state.

Catalyst	Number of atomic layers impacted by $Pd^{\delta+}$
Pd/PS	1.56
Pd/LHT	1.34
Pd/HHT	1.50
Pd/PS200	1.70
Pd/PS400	1.80
Pd/PS600	1.95
Pd/PS800	2.01
Pd/PS1000	1.70

Supplementary References

1. Knauer, M., Schuster, M. E., Su, D., Schlögl, R., Niessner, R. & Ivleva, N. P. Soot structure and reactivity analysis by raman microspectroscopy, temperature-programmed oxidation, and high-resolution transmission electron microscopy. *J. Phys. Chem. A* **113,** 13871-13880 (2009).
